# Pore-Opening and Ion-Conduction Mechanism in Channelrhodopsins C1C2, ChR2, and iChloC by Computational Electrophysiology and Constant-pH Simulations

**DOI:** 10.1021/acs.jcim.5c00356

**Published:** 2025-05-29

**Authors:** Songhwan Hwang, Tillmann Utesch, Caspar Schattenberg, Johannes Vierock, Han Sun

**Affiliations:** † Research Unit of Structural Chemistry & Computational Biophysics, 28417 Leibniz-Forschungsinstitut für Molekulare Pharmakologie, Berlin 13125, Germany; ‡ Institute of Biology, Department of Experimental Biophysics, Humboldt-Universität zu Berlin, Berlin 10115, Germany; § Neuroscience Research Centre, Charité Berlin, Berlin 10117, Germany; ∥ Institute of Chemistry, Technische Universität Berlin, Berlin 10623, Germany

## Abstract

Channelrhodopsins (ChRs) are photoreceptors that function as light-gated ion channels. Over the last two decades, they have become essential tools in optogenetics, enabling precise manipulation of neurons, neural circuits, and animal behavior through light. Although structural studies have provided important mechanistic insights into channelrhodopsins, a detailed understanding of their ion conduction mechanism and selectivity was proven to be challenging due to difficulties in experimentally resolving open-state structures. Here, we employed molecular dynamics (MD)-based computational electrophysiology and constant-pH simulations to obtain the fully open states of three different ChRs. A significant number of spontaneous K^+^, Na^+^, and Ca^2+^ permeation events were observed in the cation-conducting C1C2 and ChR2, as well as Cl^–^ permeations in the anion-conducting iChloC. Analyses of the ion binding sites and hydration profiles provided key insights into ion selectivity. Our study presents a robust computational framework for establishing the fully open-state structures of channelrhodopsins, laying the groundwork for the rational engineering of ion selectivity and conductivity.

## Graphical abstract

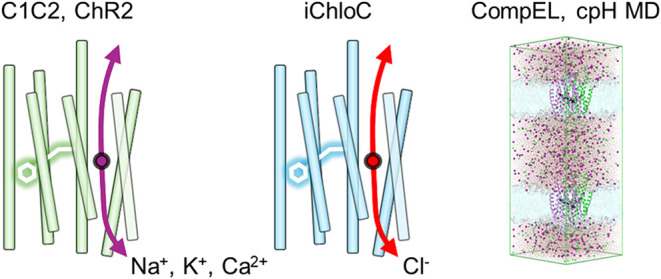

## Introduction

Channelrhodopsins are light-gated ion channels that were initially identified from the microalgae Chlamydomonas reinhardtii (C. reinhardtii).^1^ Isomerization of the light-sensitive retinylidene chromophore enables channel opening, allowing ions to flow down electrochemical gradients across the membrane. Due to their high temporal precision, channelrhodopsins have become an essential optogenetic tool for manipulating neurons and neural circuits.^2^ This has enabled the detailed study of important physiological processes such as the sleep cycle,^3^ social behavior,^4^ anxiety,^5^ pain^6^ etc. Beyond their use in basic research, channelrhodopsins have also shown promise in treating diseases like blindness,^7^ deafness,^8^ Parkinson’s disease,^9^ and epilepsy.^10^

C. reinhardtii Channelrhodopsin-1 and -2 (ChR1 and ChR2) were the first two identified natural channelrhodopsins that allowed for nonselective cation conduction.^1,11^ Over time, tremendous efforts in channel engineering and genomic screening have led to the identification of over 1000 members of the ChR family, some with enhanced ion selectivity and conductance. This includes the natural H^+^ selective Chrimson,^12^ the Na^+^ selective *Ps*ChR,^13^ K^+^ selective KCRs,^14^ and WiChR,^15^ and different anion selective channelrhodopsins (ACRs) for neuronal inhibition,^16^ engineered anion-conducting channelrhodopsins iC1C2 ^17^ and iChloC^18^ and Ca^2+^-permeable channelrhodopsins CapChRs^19^ for the photocontrol of calcium signaling.

When channelrhodopsins absorb a photon, a sequence of molecular events occurs. This so-called photocycle includes *cis*-*trans* retinal isomerization, protonation and deprotonation of the retinylidene Schiff base (Figure 1A). The intermediate states can be distinguished spectroscopically from the early conducting state by UV–vis, vibrational Fourier transform infrared spectroscopy (FTIR), and Raman spectroscopy. For instance, the late conducting state of ChR2 absorbs maximally at 520 nm, whereas the early state absorbs at 390 nm.^20^ Time-resolved FTIR and Raman spectroscopy have both also contributed to the understanding of the deprotonation and reprotonation of the retinylidene Schiff base in the channel opening.^20,21^ Notably, besides the standard C13=C14 isomerization, ChRs may undergo double isomerization at the C13=C14 and the C15=N double bonds, resulting in 13-*cis*, 15-*syn* retinal which represents the light-adapted dark state (LA_480_). Photon absorption by this state initiates a second photocycle called *syn*-photocycle with a third conducting state (N*_520_) (15-*anti* and 15-*syn*, Figure 1A).^22,23^ Compared with the *anti*-photocycle, the open state in the *syn*-photocycle exhibits smaller cation conductance but high proton selectivity.

**Figure 1:**
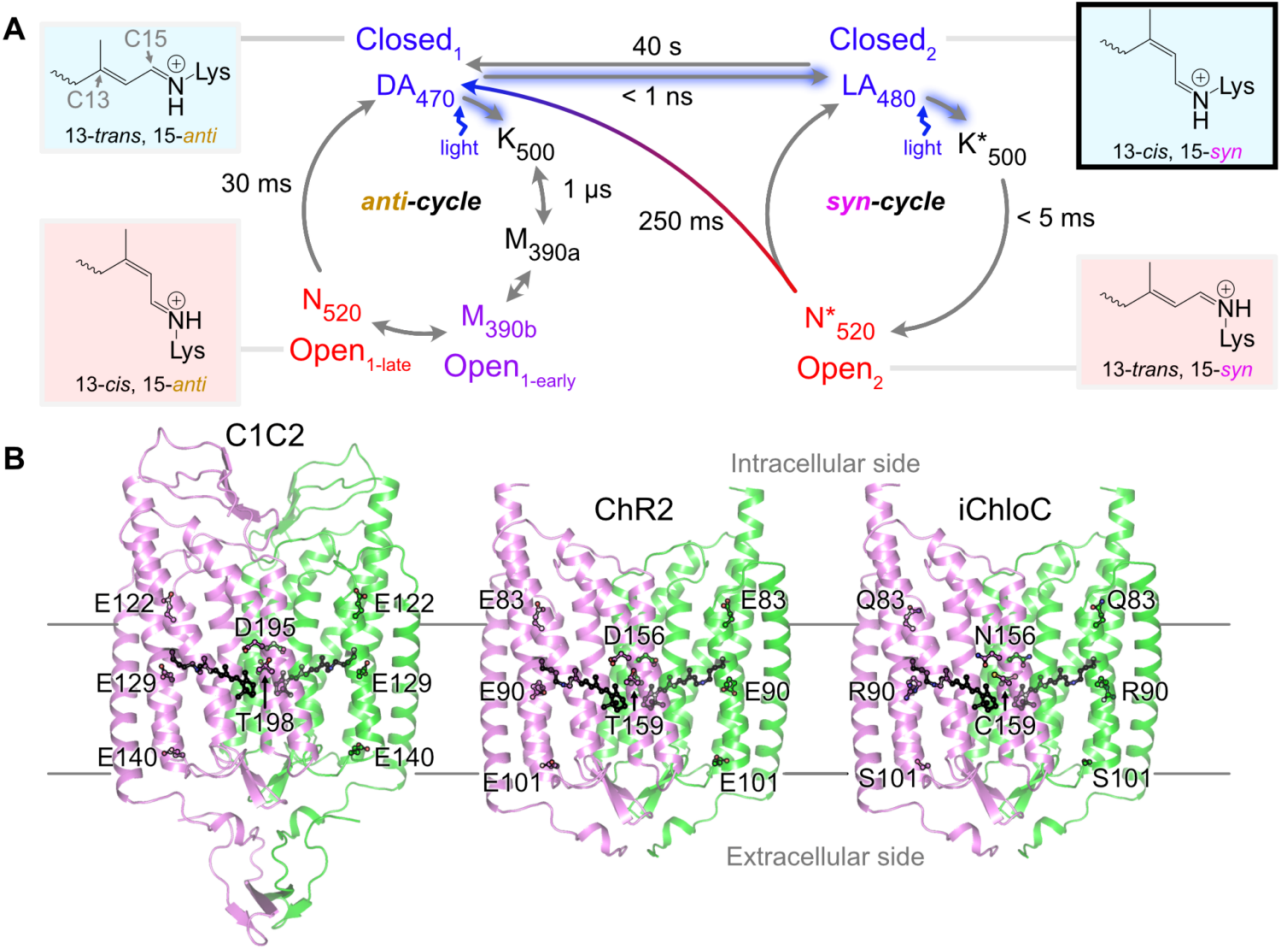
Channelrhodopsins and photocycle. (A) Photocycle of ChR2, adapted from Kuhne et al.^22^ In its dark-adapted state (DA_470_), ChR2 is in a closed state with the retinal in an all-*trans* configuration (13-*trans*, 15-*anti*). Upon light activation, two pathways are possible: (1) Within the *anti*-photocycle, where the C15=N bond is in the *anti* configuration, retinal isomerizes to 13-*cis*, 15-*anti* (K_500_) and further progresses to M_390a_ with a deprotonated retinylidene Schiff base, leading to two open states: an early open state (M_390b_) capable of H^+^ conduction and a later open state (N_520_) capable of cation conduction. (2) Alternatively, from the dark-adapted state (DA_470_), the light-adapted state with a 13-*cis*, 15-*syn* configuration (LA_480_) can be reached. This light-adapted *syn*-photocycle state undergoes K*_500_ and transitions to the open state N*_520_, enabling cation permeation. (B) Crystal structures of C1C2 (PDB entry 7E6X),^31^ ChR2 (PDB entry 6EID),^26^ and a homology model of iChloC based on the ChR2 structure. Each monomer in the homodimer is represented as a green and magenta cartoon model. The mutated residues from ChR2 to iChloC are E83Q, E90R, E101S, D156N, and T159C.

Structurally, the first X-ray structure of channelrhodopsins was resolved for an engineered chimera channel, C1C2.^24^ Later, the structural basis for a number of natural channelrhodopsins became available, including X-ray structures of ChR2,^25^ the anion-conducting GtACR1,^26^ as well as cryo-EM structures of ChRmine,^27^ bestrhodopsin,^28^ and potassium-selective HcKCR1/2.^29^ However, these structures only represent the dark-state of channelrhodopsins, which is the nonconducting state from where the photocycle starts upon illumination (Figure 1B). Very recently, the fully open-state structure of a slow-cycling mutant of HcKCR was resolved by cryo-electron microscopy (cryo-EM), following laser flash excitation.^30^ In addition, time-resolved serial femtosecond crystallography using an X-ray free electron laser provided intriguing insights into the early channel opening process without the formation of a continuous pore.^31^ A very recent study using serial synchrotron crystallographic method revealed an M_390_ intermediate state with a deprotonated 13-*cis*, 15-*anti* retinylidene Schiff base. Starting from this intermediate state and by altering the protonation state of the retinylidene Schiff base and several key acidic residues toward the open state, we successfully obtained a fully open state of wild-type C1C2 in the *anti*-photocycle by inducing transmembrane voltages in the molecular dynamics (MD) simulations.^32^

In the present study, we employed the same computational approach and performed MD-based computational electrophysiology (CompEL-MD) simulations on three types of channelrhodopsins: cation-permeable C1C2 and ChR2, and anion-conducting iChloC (Figure 1B). All simulations were initiated with the 13-*cis*, 15-*syn* configuration, representing the closed-state retinal configuration in the *syn*-photocycle. This configuration corresponds to the experimentally resolved retinal structures previously reported for the preopen and dark states of C1C2 ^31^ and ChR2.^25^ The initial aim of the study was to explore whether the channel could be opened by assigning the correct protonation states along the ion permeation pathway, even when starting from a closed-state retinal configuration. This strategy is based on the premise that retinal isomerization, which induces protonation changes in key titratable residues and subsequent channel opening, are temporally distinct events. While this approach may limit our insights into the dynamics of the chromophore, it can still offer useful information about ion permeation and channel conductance, especially in the case where the open-state chromophore structure remains experimentally elusive. Indeed, by altering the protonation state of critical acidic residues to mimic those of the open state, and by applying transmembrane voltages, we successfully sampled full transitions from the dark or preopen state to the fully open state for C1C2, ChR2, and iChloC. The simulated ion permeation pathways and conductance closely aligned with our previous simulations of C1C2 with a 13-*cis*,15-*anti* retinylidene Schiff base. Analysis of the ion binding and hydration profiles provided intriguing atomistic insights into ion selectivity. Furthermore, the protonation states of the simulated open state of C1C2 were probed using constant-pH simulations, while the effect of protonation states on the channel conductance was systematically evaluated using CompEL simulations by varying the protonation patterns.

## Results

### Channel Opening in C1C2

We performed a series of atomistic MD simulations starting from both the dark (PDB ID: 7C86) and the preopen states of C1C2 (PDB ID: 7E6X), which we embedded into a palmitoyl oleoylphosphatidylcholine (POPC) lipid bilayer (Figure S1). In the X-ray structure of the dark state of C1C2, the retinal adopts an all-*trans*, 15*-anti* configuration, while in the preopen state the authors proposed a 13-*cis*, 15-*syn* configuration,^31^ despite this isomer providing a poor fit to the experimental electron density maps. The same configuration was also observed in the closed-state X-ray structure of ChR2. As discussed earlier, spectroscopic data suggested that the 15-*syn* and 15-*anti* retinal configurations have been proposed to be involved in a branched photocycle of the channelrhodopsins (Figure 1A). In the *syn*-photocycle, the 13-*cis*, 15-*syn* configuration, however, does not correspond to a conducting state.

In the dark and photointermediate states of channelrhodopsins, protonation states of several acidic residues along the presumptive ion permeation pathway have been extensively studied previously using time-resolved infrared and Raman spectroscopy, electrophysiology in combination with mutagenesis.^21,33–35^ These residues include E122 at the inner gate, E129 of the central gate, D195 as part of the DC pair, and one of the counterions, D292 (Figure 2A). In our simulations, we defined the protonation state of these residues in both dark- and open-state simulations based on generally accepted experimental evidence (Table S1). For the preopen state structure, we biased the simulations by adopting the protonation state of the open state. However, it should be noted that ambiguity remains regarding the protonation states of these titratable residues, especially the two counterions, E162 and D292.^36^ In our initial round of simulations, we assumed that both counterions were deprotonated in the dark state, while D292 was modeled as protonated in the preopen state.

**Figure 2:**
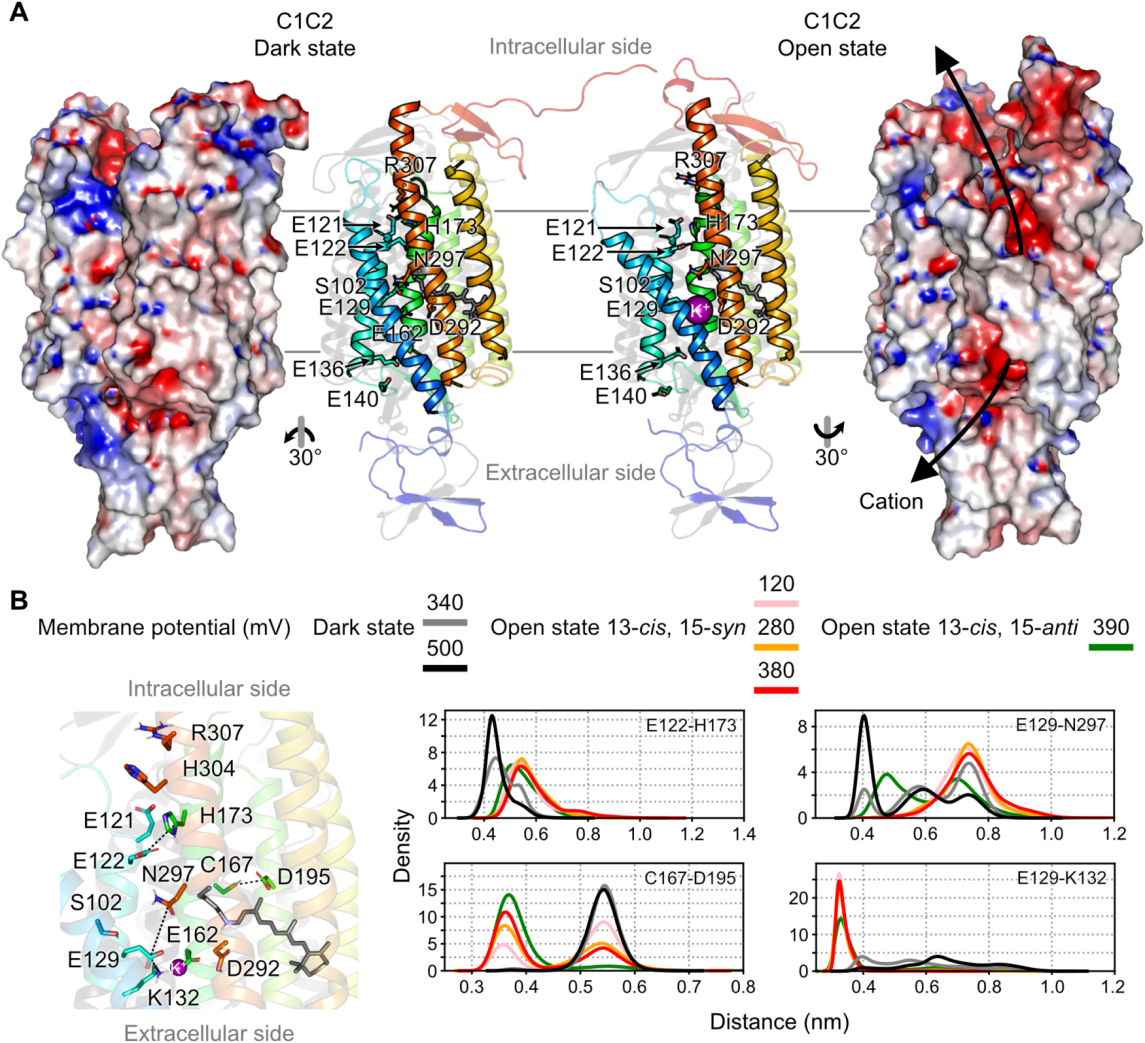
Dark and open states of C1C2. (A) The dark and open states of C1C2 are depicted as an electrostatic potential map and cartoon representation. Key residues involved in the cation conduction pathway (E122, E129, E136, E140, and E162) are highlighted as sticks. The shown exemplary structures represent the final snapshot from one simulation run. (B) Comparison of the distance distributions of specific residue pairs between the dark state at −340 and −500 mV (gray and black, respectively) and the open states with 13-*cis*, 15-*syn* retinal at −120, −280, and −380 mV (pink, orange, and red, respectively) and 13-*cis*, 15-*anti* retinal at −390 mV (green).^32^ All distance distributions were calculated for monomer A of inward-permeating channels based on five 2-μs simulation replicas. For the distance calculations, the following side-chain atoms were considered: Cys-Sγ, Asp-Cγ, Glu-Cδ, His-Cγ, Lys-Nζ, and Asn-Cγ.

Starting from the dark and preopen conformations of C1C2, respectively, we conducted five 2-μs runs under transmembrane voltages ranging from ±120 to ±500 mV (Table S2). In all simulations starting from the preopen conformation with a 13-*cis*, 15-*syn* retinal, we observed the formation of a continuous water pore (Figure S2) and the gradual opening of both intracellular and extracellular gates (Figure 2). In contrast, simulations starting from the dark state showed that the central and intracellular gates remained closed, with the extracellular and intracellular cavities being more dehydrated compared to the open ones. No single-cation permeation was observed in the dark-state simulations (Figure S3). In open-state simulations, the most significant pore-opening dynamics were observed at higher transmembrane voltages (Figure 2). These high voltages, although beyond physiological conditions, were employed to accelerate channel opening and ion conduction during the MD simulations, a commonly used approach for simulating ion channels with low conductance^30,37–39^ and voltage-sensitivity in genetically encoded voltage indicators.^40^ Throughout the MD-based computational electrophysiology (CompEL-MD) simulations, the channel structures remained stable (Figure S4). Compared to those in the dark state, significant conformational changes and helix tilting were observed in the open state, particularly in transmembrane helices (TM) 1 and 7. Here, a pore with negative electrostatic environment formed between TM1–3 and TM7 (Figure 2A). Particularly, we identified several key residue-pair distances that differ substantially between dark- and open-state simulations in three regions: (i) the inner gate, E122–H173; (ii) the DC pair, C167–D195; (iii) the central gate, E129–K132 and E129–N297 (Figure 2B). We further compared these key differences between the open-state simulations from this study, which started with a 13-*cis*, 15-*syn* retinal, and our previous simulations with a 13-*cis*, 15-*anti*.^32^ Notably, the two open states exhibited a high structural similarity (Figure 2B).

### K^+^, Na^+^, and Ca^2+^ Permeation and Hydration in C1C2

After the pore of the C1C2 opened during the simulations, K^+^ permeation was observed across all five simulation runs at ±380 mV. The number of ion permeation events varied between 1 and 24 across the replicas (Figure 3, Table S2), corresponding to conductances from 0.04 to 1.01 pS. The simulated conductance for the 13-*cis*, 15-*syn* closely aligns with our previous simulations of the 13-*cis*, 15-*anti* C1C2. Simulations conducted at −380 and −280 mV showed substantially higher conductances compared to those at −120 mV within the current time scale (Figure 3B). This discrepancy may be due to the shorter time acquired to achieve channel opening at stronger transmembrane voltages compared with the weaker ones. We further performed two additional 1 μs simulation replicas starting from an open-state snapshot, where ion conduction had been observed. These simulations did not show a significant increase in conductance but did reveal a few outward permeation events (Figure S5).

**Figure 3:**
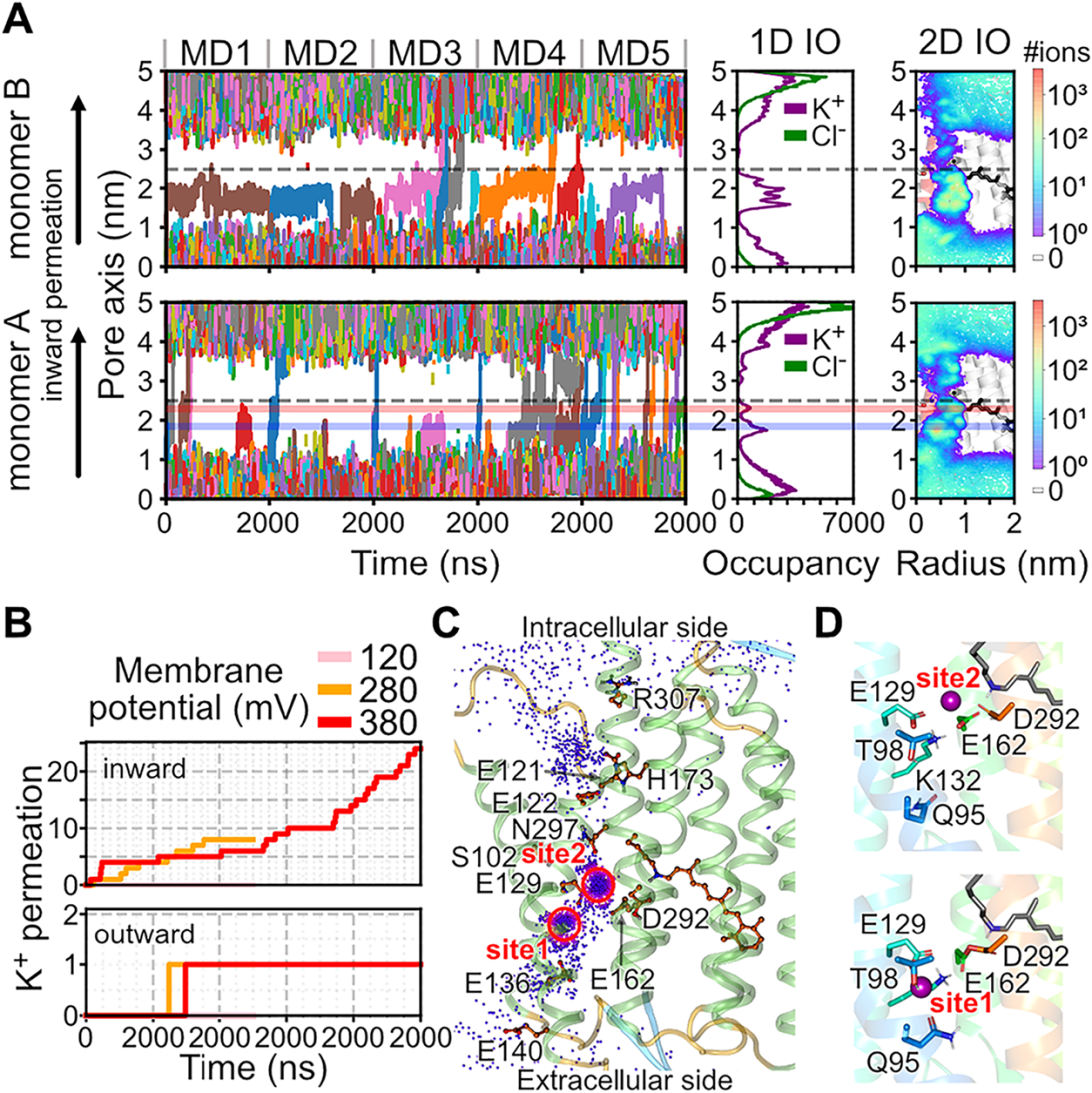
Cation permeation in open-state C1C2. (A) Inward K^+^ permeation through the pore of C1C2 at −380 mV. (left) Traces of K^+^ passing through the pore represented by a cylinder with a 2 nm radius and 5 nm height, centered at the Cα position of the S102 (gray dotted lines). (Middle) Cumulative one-dimensional K^+^ (purple) and Cl^–^ (green) occupancy along the pore axis. (Right) 2D K^+^ density within the pore region, mapped onto an MD snapshot of open-state C1C2, with the protonated retinylidene Schiff base depicted as a black stick model. Two major K^+^ binding sites, site 1 and site 2, are indicated by semitransparent lines in blue and red, respectively. (B) Cumulative number of inward and outward K^+^ permeation events passing through the C1C2. (C) Overlaid positions of K^+^ ions from a 2-μs MD trajectory at −380 mV, depicted as purple dots on an MD snapshot of the open-state C1C2. (D) Two major K^+^ binding sites of C1C2. Ion binding residues are represented as sticks, and K^+^ ions are presented as purple spheres.

In the simulations, K^+^ permeated through the interfaces of the helices TM1–3 and TM7, consistent with the ion permeation pathway previously proposed by structural analyses,^24,25^ functional studies,^41^ and our earlier simulations with a 13-*cis*, 15-*anti* retinal configuration.^32^ Specifically, during ion permeation, four conserved glutamates in TM2 (E122, E129, E136, and E140) interacted with K^+^ ions (Figure 3C). By tracking the position of K^+^ ions along the pore, we identified two major ion binding sites in the pore region close to the central gate (Figure 3D). At site 1, Q95, T98, E129, and E162 contribute to K^+^ binding, while at site 2 ion binding mainly involves the acidic residues E129, E162, and D292. These binding sites again align closely with the results obtained from our previous simulations of the 13-*cis*, 15-*anti* in C1C2.^32^

Experimentally, C1C2 exhibits no selectivity between Na^+^ and K^+^,^17^ while its conductance for Ca^2+^ is significantly lower.^19^ To investigate the conduction of different cation types, we performed five 2-μs simulations with either NaCl or CaCl_2_, starting from a snapshot of the inward-permeating open state with a 13-*cis*, 15-*anti* retinal configuration from our earlier study, which had focused exclusively on K^+^ permeation.^32^ The switch to the 13-*cis*, 15-*anti* configuration here was also motivated by the fact that calcium conductivity has predominantly been studied experimentally in the context of the *anti*-cycle. For these simulations, we employed a multisite Ca^2+^ parameter,^42^ which has been extensively verified on the ion permeation simulations of AMPA receptor.^43^ Our simulations on C1C2 yielded a Na^+^ conductance of 1.9 pS at −370 mV (Figure S6A, Table S2), while the Ca^2+^ conductance was notably lower at 0.1 pS under the same voltage, compared to the K^+^ conductance of 1.0 pS at −380 mV (Figure S6C, Table S2). Analysis of the ion binding sites indicated that the reduced Ca^2+^ conductance is mostly due to an additional site by E136 and E140 referred to as site 0. Ca^2+^ ions occupy this site for a much longer time compared to monovalent ions, which may explain the reduced Ca^2+^ conductance. Notably, the binding sites 1 and 2 identified for K^+^ and Na^+^ were also present for Ca^2+^ (Figure S6B,D).

Furthermore, we analyzed the hydration levels of the K^+^ and Na^+^ ions during permeation (Figure 4). In the bulk solvent, the number of water molecules in the first hydration shell was 6–7 for K^+^ and 5–6 for Na^+^. In contrast, within the pore region, the water coordination number of both ions decreased to 4–5 due to transient interactions with acidic residues E136 and E140 on the extracellular side when entering the pore. These findings suggest that K^+^ and Na^+^ ions permeate through the channel pore in a partially dehydrated state.

**Figure 4:**
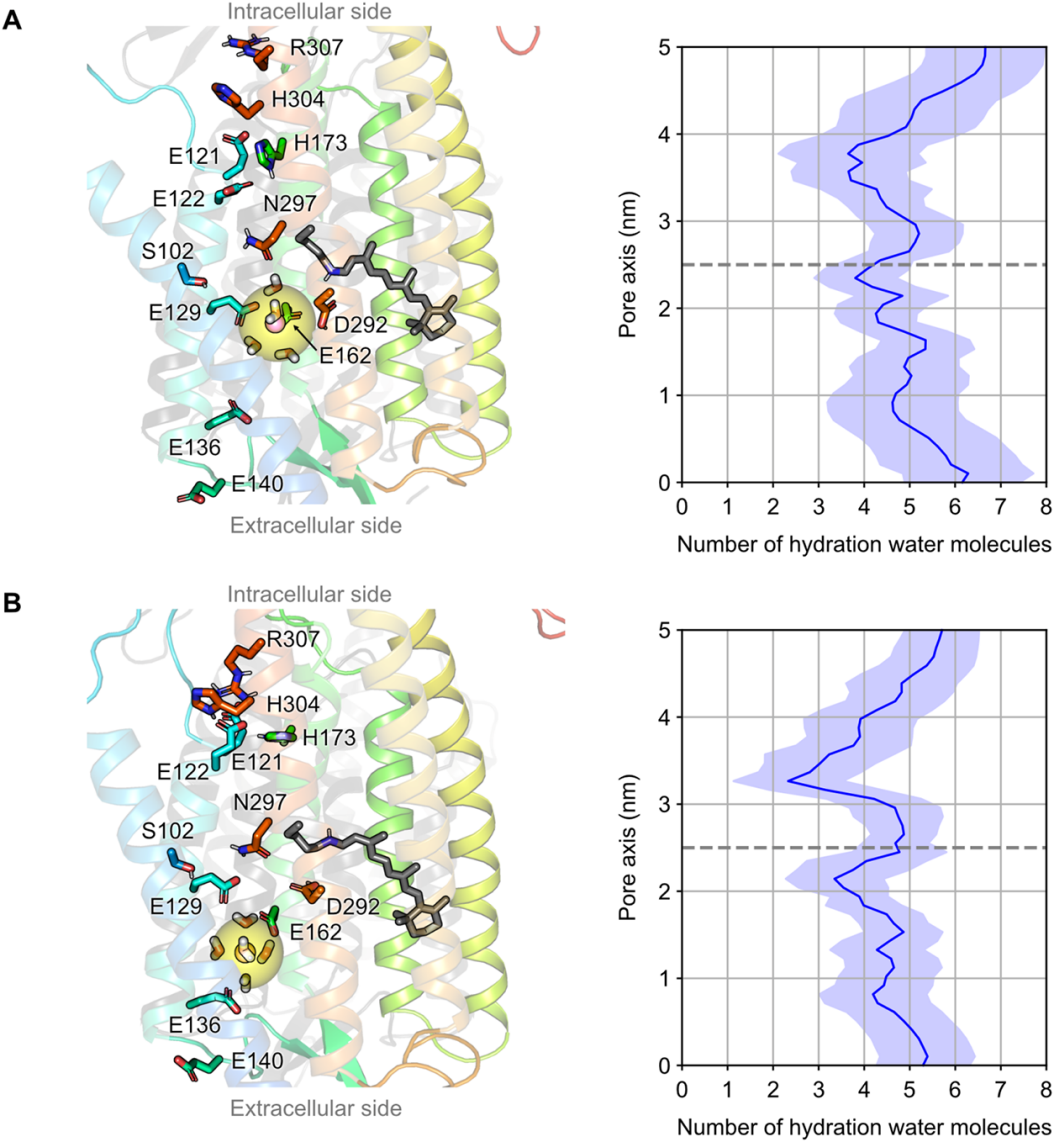
Hydration profiles of K^+^ and Na^+^ during permeation in open-state C1C2. (Left) Water molecules within (A) a 3.5 Å sphere centered on K^+^ and (B) a 3.2 Å sphere centered on Na^+^. Note that the reduced water coordination around the ions is compensated by interactions with E129 and E162. (A, right) The number of coordinating waters in the first hydration shell of K^+^ during inward permeation under −380 mV, calculated from five 2-μs replicas. (B, right) Hydration profile of Na^+^ during inward permeation under −370 mV, calculated from five 2-μs replicas. Average hydration levels are shown as blue solid lines with standard deviations represented by blue shaded regions.

### Channel Opening and Ion Permeation in ChR2

Next, we investigated channel opening and ion permeation in the most widely used light-gated channelrhodopsin, ChR2. The dark-state X-ray structure of ChR2 (PDB ID: 6EID) exhibited a mixed conformational state, comprising both all-*trans*, 15-*anti* and 13-*cis*, 15-*syn* configurations.^25^ For the open-state simulations of ChR2, we selected the same 13-*cis*, 15-*syn* configuration with a protonated retinylidene Schiff base as used in the simulations of C1C2, but the overall structure of ChR2 at the starting point remained closed. To initiate channel opening, we biased the simulations by adjusting the protonation states of titratable residues along the ion conduction pathway based on the experimental evidence, similar to the open-state simulations of C1C2 (Table S1). Applying a transmembrane voltage of −480 mV successfully opens the channel for K^+^ in most of the five 2-μs simulation runs (Figure 5A,B). Similar to the case for C1C2, these simulations revealed strong inward rectification (Figure 5C). The inward conductance was calculated as 0.60 ± 0.35 pS, which fits to the very low conductance of N520* in ChR2 from patch-clamp electrophysiology.^44^ From ChR2, we observed both inward and outward K^+^ permeations, with significantly higher inward K^+^ conduction (Figure S7). The ion conduction pathway, major ion binding sites within the pore, and pore opening in ChR2 are highly comparable to those in C1C2 (Figures 5D and S8). Four glutamate residues (E83, E90, E97, and E101) in TM2 interacted with K^+^ during conduction. As in C1C2, two major K^+^ binding sites were identified in the central gate region: at site 1, K^+^ ions were coordinated by Q56 and E90, while at site 2, E90, E123, and D253 coordinate K^+^ (Figure 5E).

**Figure 5:**
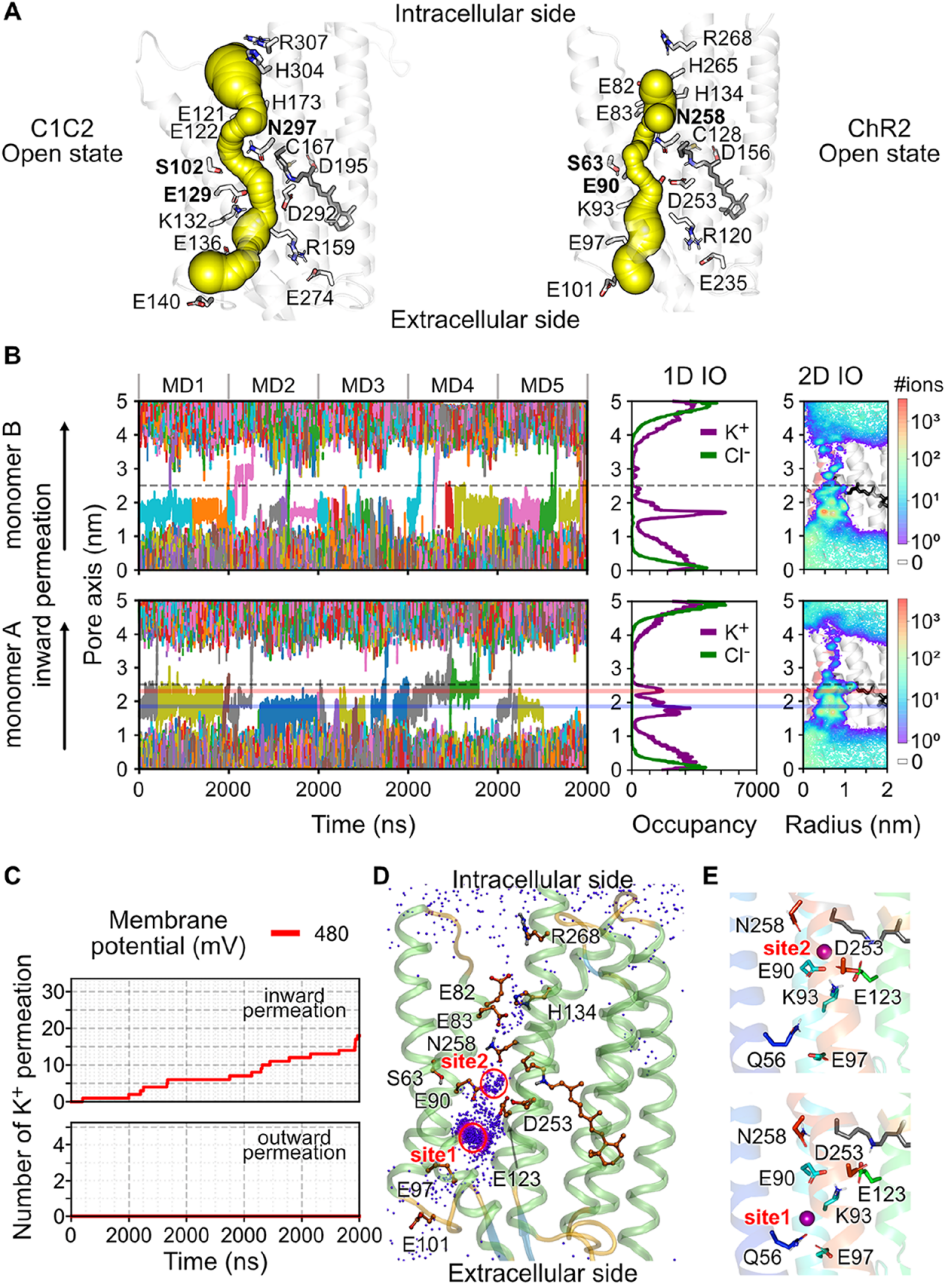
Pore opening and cation permeation in open-state ChR2. (A) Comparison of the channel pore in open-state C1C2 and ChR2 using CAVER 3.0.^73^ Predicted tunnels are depicted as spheres. (B) Inward K^+^ permeation through ChR2 under −480 mV. (Left) Traces of K^+^ ions passing through the ChR2 pore represented by a cylinder with a 2 nm radius and 5 nm height, centered at the Cα position of S63 (gray dotted lines). (Middle) Cumulative one-dimensional K^+^ (purple) and Cl^–^ (green) occupancy along the pore axis. (Right) 2D K^+^ density within the pore region, mapped onto an MD snapshot of open-state ChR2. The protonated retinylidene Schiff base is depicted as a black stick model. Two major K^+^ binding sites, site 1 and site 2, are indicated by semitransparent lines in blue and red, respectively. (C) Cumulative number of inward and outward K^+^ permeation events passing through the central gate of ChR2. (D) Position of K^+^ ions from five 2-μs MD runs under −480 mV, depicted as purple dots, mapped onto an MD snapshot of the open-state ChR2. (E) Two major K^+^ ion binding sites in ChR2. Ion binding residues are represented as stick models, and K^+^ ions are shown as purple spheres.

### Anion Binding and Selectivity of iChloC

To create Cl^–^ permeable channelrhodopsins, some of the electronegative residues of ChR2 along the ion conduction pathway have been replaced with neutral or electropositive ones. As a result, channelrhodopsins such as iChloC have been developed with the following mutations: E83Q, E90R, E101S, D156N, T159C.^18^ To gain a better understanding of how channelrhodopsins achieve ion selectivity, CompEL-MD simulations in 600 mM KCl were conducted. The structural model of iChloC was generated based on the crystal structure of ChR2 (PDB ID: 6EID) and contains 13-*cis*, 15-*syn* retinal. During five runs of 2-μs simulations at ± 560 mV, we did not observe any ion permeation events in iChloC. This stands in contrast to the simulations of C1C2 and ChR2, where under similar conditions a significant number of ion permeation events were observed. Additionally, 2 runs of 4-μs at higher voltages (±770 mV) were performed, showing only 3 inward and 3 outward Cl^–^ permeations, respectively (Figure 6B,C), indicating very low conductance property of iChloC. Analysis of ion binding profiles suggested that Cl^–^ occupied the pore region in iChloC. Above and below the central gate, two stable Cl^–^ binding sites were identified, which are located close to the cation binding sites in C1C2 and ChR2. The first Cl^–^ binding site involves R120, K93, and R90, with R90 being one of the key mutated residues in the engineering of cation-conducting channelrhodopsins (E90 in ChR2) into anion variants. Binding of the second Cl^–^ binding site is mainly defined by Q83, which has been mutated from E83 in ChR2 (Figure 6D,E). Taken together, the low Cl^–^ conductivity of iChloC may be attributed to the long dwell times at its major binding sites, similarly to the reduced conductance observed for Ca^2+^ due to its stable binding at site 0.

**Figure 6:**
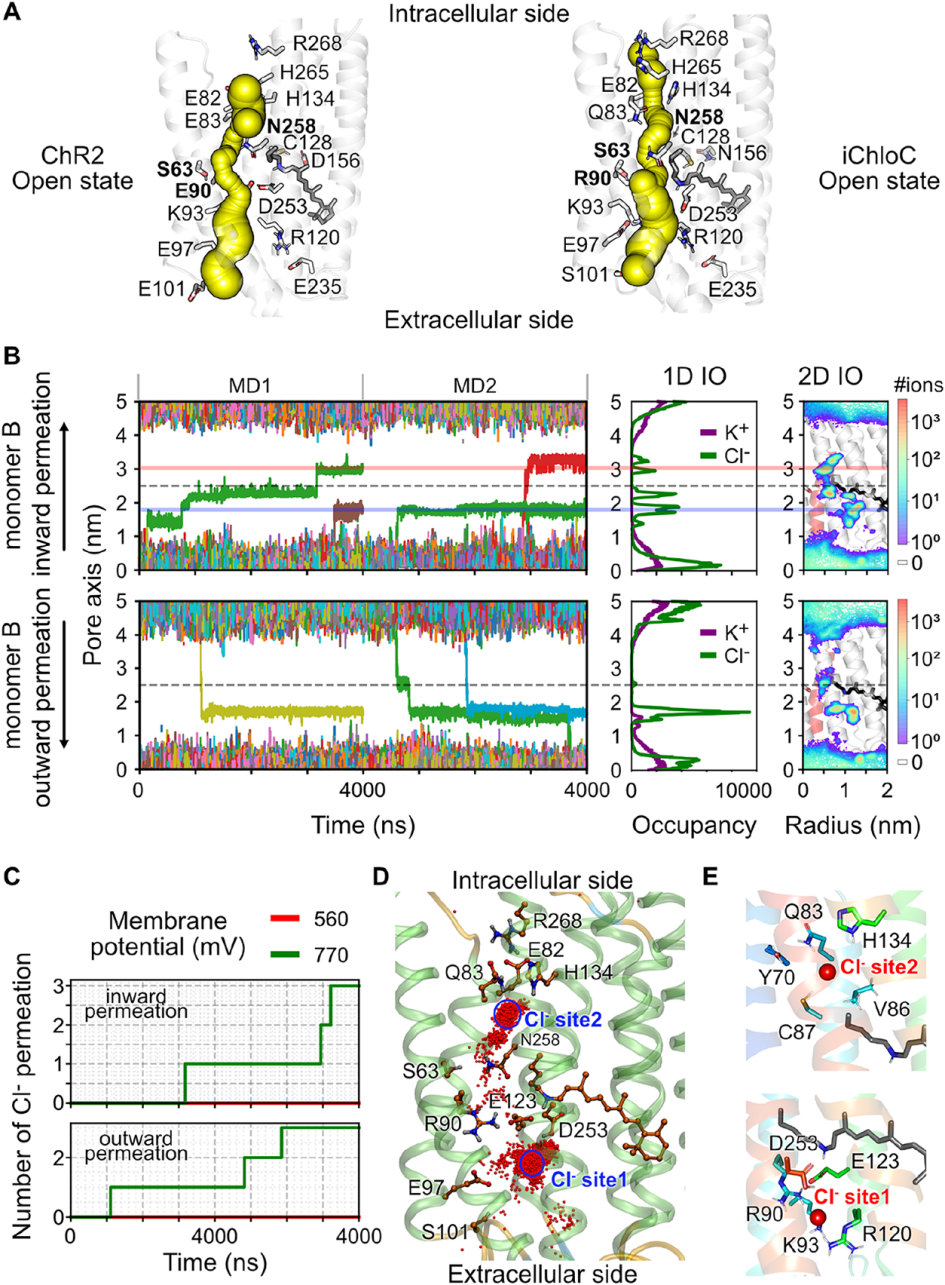
Pore opening and Cl^–^ permeation in the open-state iChloC. (A) Comparison of the channel pores from MD snapshots of the open-state ChR2 and iChloC using CAVER 3.0.^73^ Predicted tunnels are shown as spheres. (B) Inward and outward Cl^–^ permeation through the iChloC under ± 770 mV. (Left) Traces of Cl^–^ passing through the pore of iChloC represented by a cylinder with a 2 nm radius and 5 nm height, centered at the Cα position of S63 (gray dotted lines). (Middle) Cumulative one-dimensional K^+^ (purple) and Cl^–^ (green) occupancy along the pore axis. (Right) 2D Cl^–^ density within the pore region, mapped onto an MD snapshot of the open-state iChloC, with the protonated retinylidene Schiff base depicted as a black stick model. Two major Cl^–^ binding sites, Cl^–^ site 1 and Cl^–^ site 2, are indicated by semitransparent lines in blue and red, respectively. (C) Cumulative number of inward and outward Cl^–^ permeation events through the central gate of iChloC. (D) Positions of Cl^–^ ions derived from a 4-μs MD under +770 mV, depicted as red dots mapped onto an exemplary snapshot of the open-state iChloC. (E) Two major Cl^–^ binding sites of iChloC. Ion binding residues are represented as stick models, and Cl^–^ ions are shown as red spheres.

### Probing Protonation States of the Open-State C1C2

We further probed the protonation states of the titratable residues in the dark and open states of C1C2 using constant-pH MD (cpH-MD) simulations. Different from conventional MD simulations, where the protonation states of titratable residues remain fixed, cpH-MD simulations allow the protonation state of a titratable residue to dynamically change according to the local electrostatic environment and the pH of the solution.^45^ Here, we combined the cpH-MD setup with CompEL-MD to induce a transmembrane potential ranging from −340 to −380 mV. It is important to note that the initial structures for the cpH-MD simulations were selected from a final snapshot of the aforementioned ion permeation simulations, in which the two subunits exhibit asymmetric degrees of channel opening. As a result, the protonation states in each subunit may differ due to this structural asymmetry.

In C1C2, variations in the protonation states were primarily observed in five residues along the ion permeation pathway. These residues are (i) E122 in TM2; (ii) E129 in TM2; (iii) E162 in TM3; (iv) D195 in TM4; and (v) D292 in TM7. Interestingly, both E129 and D195 remained consistently protonated in the dark state but showed a tendency toward deprotonation in the open-state simulations. D292 remained deprotonated in all states, while for E122 and E162 the protonation prediction was less conclusive, as it varied depending on the specific subunit examined (Figure 7, Figure S9). During the CpH-MD simulations, monomer B showed a higher tendency to revert to a nonconductive state, as indicated by a protonation pattern that more closely resembled the dark state (Figure 7) and a decrease in ion densities within this monomer compared to monomer A. The protonation states of all other titratable residues including all aspartic acids, glutamic acids, and histidines did not significantly differ between the closed and open states. Histidines in the ion permeation pathway were predicted to be singly protonated on the Nε position.

**Figure 7:**
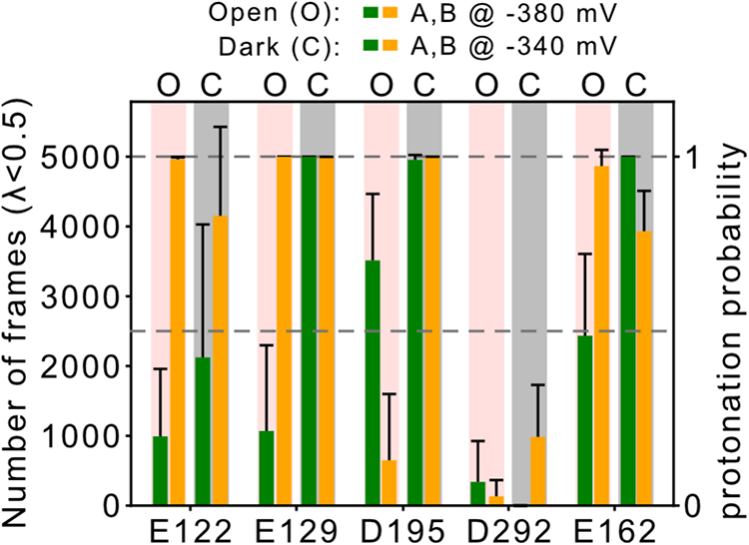
Probing protonation states of key titratable residues during ion permeation using cpH-MD simulations. Titratable residues in the inner gate (E122), central gate (E129), DC pair (D195), and counterions (D292 and E162) exhibited variations in their protonation states during cpH-MD simulations. Starting structures were selected from the final snapshots of CompEL-MD simulations of the dark and open states of C1C2, respectively. The total occurrences of a protonated residue were summed across 5000 frames for the open state (O or red shaded) and dark state (closed, C or gray shaded), separately for each subunit (monomer A: green, monomer B: yellow). Black bars represent the standard deviation of the averages obtained from three independent 500 ns runs. The continuous reaction coordinate λ indicates the protonation state of the titratable residues. While λ = 0 indicates a protonated state, λ = 1 corresponds to the deprotonated state for the shown acidic residues. This underlying energy function defines the behavior of the intermediate states in this continuous model.

To further address the ambiguity surrounding the protonation states of D292 and E162, we conducted a series of CompEL-MD simulations with variation in their protonation states, as summarized in Table S3 and Figure S10. First, we protonated the alternative counterion, E162, where the simulations revealed a 5-fold reduction in K^+^ conductance compared to the case where D292 was protonated. Furthermore, the simultaneous protonation of both E162 and D292 resulted in an even more pronounced reduction in ion conductivity, decreasing by a factor of 12. Conversely, deprotonation of both D292 and E162 resulted in a 1.8-fold increase in inward conductance and a significant enhancement in outward permeation (1.43 pS). Given the importance of D195 protonation in the transition to the dark state, we extended our simulations to explore how protonation at D195 and D292 impacts ion conductivity. Interestingly, inward ion conduction was still observed, albeit with a 2-fold decrease relative to the simulations where only D292 was protonated. Overall, the comparison of the conductance across different protonation states demonstrated that deprotonation of all three residues—E162, D195, and D292—increases cation conductance. Among these, the deprotonation of E162 was shown to be particularly crucial for maintaining relatively high conductance in the simulations.

## Discussion

Channelrhodopsins are well-established optogenetic tools for the noninvasive control of neuronal excitability. Despite their extensive use in studying neuronal networks and animal behavior, the conventional structural biology of channelrhodopsins has been largely limited to the dark state, corresponding to the closed-state conformation. Recently, time-resolved serial crystallography has opened the possibility of monitoring structural changes during the photocycle following light illumination. However, two time-resolved serial crystallography studies on C1C2 revealed that the transition from the M_390_ intermediate to the N_520_ open state is hindered in the crystal.^31,32^ As a result, the M_390_ state remains the most open structure of C1C2 that has been experimentally resolved within the photocycle. According to electrophysiological characterization, the M_390b_ state (Open_1‑early_) is proton-conducting but not permeable to alkali cations.^22,46^

In a very recent study, we started from the M_390b_ intermediate state and successfully obtained a fully open state of C1C2 in the *anti*-photocycle by MD simulations.^32^ This was achieved by adjusting the protonation state of the retinylidene Schiff base and nearby acidic residues toward the open state under applied transmembrane voltages. The simulated absorption differences between the open N_520_ state, the M_390b_ intermediate state, and the dark state showed excellent agreement with experimental absorption spectra. These results further validated the open-state structure derived from our MD simulations. It should be noted that this approach differs conceptually from previous MD studies, which primarily focused on the formation of water pores without fully opening the channel to permit cation conduction.^33,47^ Although some recent computational studies have constructed potential mean force (PMF) profiles for ion conduction,^48,49^ these studies rely on biased simulations, which differs from our approach. Nevertheless, these studies offer critical insights into the energy landscape and the role of mutations on the conductance mechanism of cations.

In this study, we employed MD-based computational electrophysiology to simulate three types of channelrhodopsins with a 13-*cis*,15-*syn* retinal configuration, which corresponds to the experimentally resolved configuration in the *syn*-photocycle.^25,31^ According to spectroscopic and electrophysiology data, this configuration typically represents a nonconducting state. However, by biasing the protonation states of the retinylidene Schiff base and nearby acidic residues, we were able to fully open the channel. This resulted in an ion permeation pathway and conformational changes in the channel that were nearly identical to those observed in the 13-*cis*,15-*anti* configuration. These findings suggest that by simulating under transmembrane voltages and introducing protonation biases reflective of the open state, channelrhodopsins can be reliably opened even when the retinylidene Schiff base adopts a nonconducting state configuration. By demonstrating this approach across three different channelrhodopsins, we propose that this method could be routinely applied to other channelrhodopsins to investigate their ion conduction mechanisms, especially in cases where the open-state chromophore is challenging to resolve using experimental means. Nonetheless, we also acknowledge that the *syn*- and *anti*-photocycles exhibit distinct cation conductivity properties in experimental studies. Our simulation approach was not able to capture or explain these differences.

Capturing the complete conformational transition between the open and closed states during unbiased MD simulations is typically challenging for most ion channels, often requiring the use of enhanced sampling techniques. However, in channelrhodopsins like ChR2, a full transition from closed to open can be achieved by applying a transmembrane potential and biasing the protonation states during microsecond-scale simulations. This is primarily because the conformational changes in the inner and central gates are relatively small and localized, mediated by protonation changes.

During K^+^ conduction, the ion density within the pores of C1C2 and ChR2 is very low. Typically, we observed a single-cation conduction mechanism (Movie S1), which may help explain the experimentally characterized low conductivities of these channelrhodopsins. Notably, the single-cation conduction mechanism differs substantially from that of other bacterial and animal cation channels, where multiple cations permeated sequentially via knock-on mechanisms.^37,39,50^ To the best of our knowledge, the only cation channel known to share a similar single-cation conduction mechanism is the lysosomal K^+^ channel TMEM175 based on MD simulations.^51^ Notably, like channelrhodopsins, TMEM175 conducts both K^+^ and H^+^.^52^ Therefore, we propose that the single-ion conduction mechanism may be advantageous for the simultaneous transport of alkali metals and protons.

As discussed in the introduction, electrophysiology measurements have shown that the *syn*-photocycle exhibits higher proton selectivity compared to the *anti*-photocycle.^22,23^ While our computational approach, based on classical MD simulations, cannot directly address proton selectivity, the open-state structures suggested by the MD simulations may provide a valuable foundation for future quantum mechanics/molecular mechanics (QM/MM) MD studies at probing the proton conduction mechanism.

Achieving and understanding high ion selectivity, such as K^+^ or Ca^2+^ selectivity, remain cutting-edge research directions within the channelrhodopsin community. Our simulations of cation-permeable C1C2 and ChR2 revealed a contrasting ion selectivity compared to the anion-channelrhodopsin iChloC, primarily due to the negative electrostatic environment of the pore. This ion-selectivity mechanism differs from most other ion channels, where selectivity is governed by a specific region known as the selectivity filter. Previous MD simulations have suggested that the high selectivity of K^+^ channels arises from the complete dehydration of several K^+^ within the selectivity filter.^53^ In contrast, the K^+^ channel TMEM175, which lacks a canonical selectivity filter, achieves a degree of K^+^ selectivity through a narrow hydrophobic constriction site.^51^ This site is more difficult for Na^+^ to traverse than K^+^ due to the higher dehydration energy of Na^+^. In this study, we calculated the hydration levels of K^+^ and Na^+^ ions within the pore and found that both K^+^ and Na^+^ ions remained partially hydrated during permeation, even at the constriction sites. This result aligns with the previous MD study on C1C2,^49^ where partial dehydration of Na^+^ and Ca^2+^ was observed. Similar observation was also made on other nonselective cation channels, where K^+^ and Na^+^ are either both partially hydrated,^39,54^ or structural plasticity in the selectivity filter allows for the passage of both dehydrated K^+^ and hydrated Na^+^.^55^ In conclusion, the partially hydrated levels of K^+^ and Na^+^ align with the cation nonselective nature of C1C2 and ChR2.

MD simulations of C1C2 and ChR2 revealed a strong inward-rectifying property, particularly when the simulations started from the preopen and closed-state structures. Reduced inward rectification was observed when the simulations began directly from the open-state structure. This computational observation aligns well with electrophysiology measurements of C1C2 and ChR2, which exhibit nonlinear current–voltage relationships in the early conducting state.^56^ This behavior has been attributed to the presence of a strong external cation binding site, which was also observed in our simulations. Furthermore, we observed drastically reduced Ca^2+^ permeation in comparison to that of monovalent cations. This finding resulted in strong binding of Ca^2+^ to the extracellular site formed by E136 and E140 which was only visited transiently by Na^+^ and K^+^. Notably, a recent computational study by Prignano et al.^49^ also predicted a higher energy barrier along the ion conduction pathway for Ca^2+^ compared to Na^+^.

To further explore the protonation state of open-state C1C2, we employed two different computational approaches. First, we used a constant-pH simulation setup to probe the protonation state of standard titratable residues in the open state of C1C2. In these simulations, we observed a tendency for E129 and D195 to deprotonate in the open state, consistent with experimental findings.^20,22,35^ However, the protonation tendencies of E122 and E162 remained inconclusive. To investigate how the protonation states of the two counterions (D292 and E162) and D195 affect cation conduction, we systematically varied their protonation states across several CompEL-MD simulations. Notably, the highest K^+^ conductance was observed when all three of these acidic residues were deprotonated. It should be noted that a recent study by Prignano et al. using a complementary approach including metadynamics simulations, PMF calculations, and electrophysiology experiments revealed that D195 was protonated in the *syn*-photocycle. In our CompEL-MD simulatons, protonation of D195, however, led to reduced conductance.

In conclusion, this study presents a computational strategy for simulating the complete transition from the closed state to the open state in channelrhodopsins. We demonstrated this approach using three different channelrhodopsins and systematically compared the results with the previous electrophysiological data. The insights gained from MD simulations offer a detailed understanding of channel opening, ion conduction mechanism, and selectivity, paving the way for the rational engineering of new channelrhodopsin variants with enhanced ion selectivity and conductance.

## Materials and Methods

### Molecular Dynamics Simulations

All atomistic MD simulations were performed with Gromacs 2019.3 and 2019.6,^57^ with the CHARMM36m force field,^58^ and previously published force field parameters of the retinylidene Schiff base.^59^ The simulation system was prepared using CHARMM-GUI^60^ based on the homodimeric structure of C1C2 (PDB: 7C86 and 7E6X), ChR2 (6EID), and iChloC. The missing parts of C1C2 (Q112-T117, G329, G330) were modeled, and mutations (E83Q, E90R, E101S, D156N, T159C) for iChloC based on the ChR2 structure were introduced using Modeller 10.^61^ The protein was embedded into 1-palmitoyl-2-oleoyl-*sn*-glycero-3-phosphocholine (POPC) lipids and solvated with 600 mM KCl, NaCl, or CaCl_2_ and TIP3 water models.^62^ K^+^, Na^+^, Cl^–^ ions were modeled with standard parameters, while Ca^2+^ ion was modeled with the multisite parameter.^42^ The details of the system are described in Tables S1–S3. The system was energy-minimized with the steepest descent method and equilibrated with stepwise release of positional restraints in an isothermal–isobaric (NPT) ensemble (Table S4). Temperature control at 303 K and pressure control at 1 bar were carried out using the Velocity-rescaling thermostat^63^ and the Berendsen^64^ or Parrinello–Rahman barostats,^65^ respectively.

For CompEL-MD,^66^ the equilibrated systems were stacked along the normal axis, resulting in a double lipid bilayer system (Figure S1). The two compartments were defined by the center of mass of the central gate residues (C1C2: S102, E129, N297; ChR2: S63, E90, N258, iChloC: S63, R90, and N258) of the dimer, allowing us to introduce ion concentration imbalance and generate membrane potential (Tables S2–S3). To monitor and maintain the charge imbalance between the two compartments, we defined a virtual cylinder centered on the defined group with a radius of 3 nm and upper and lower heights of 1.5 and 1 nm, respectively. To maintain the charge imbalance throughout the simulations, we used the deterministic protocol in the CompEL-MD. This double lipid bilayer system was energy-minimized. Production runs of 2 μs in the NPT ensemble were repeated five times for statistical analysis. During the MD simulations, the short-range cutoff for electrostatic and van der Waals interactions were set to 1.2 nm. For long-range electrostatic interactions, the particle mesh Ewald method^67^ was used. The simulations were conducted with periodic boundary conditions, and hydrogens attached to heteroatoms were constrained using the LINCS algorithm.^68^

CpH-MD simulations were performed using the Gromacs 2021 β version,^45^ with CHARMM36m force field.^58^ We used the β-version of GROMACS, because the cpH algorithms were not part of the official GROMACS release when we started the calculations. Starting structures were selected from the final snapshots of CompEL-MD simulations of the dark state at −340 mV and the open state at −380 mV. The systems were energy-minimized using the steepest decent method and equilibrated in the NPT ensemble at 303 K and 1 bar, using the Parrinello–Rahman barostat.^65^ Subsequently, three independent 500 ns cpH-MD were conducted. A cutoff of 1.2 nm was applied for short-range van der Waals and electrostatic interactions, while long-range electrostatic interactions were treated using the particle mesh Ewald method.^67^ Bond constraints were applied using the LINCS algorithm.^68^

### Analysis of the MD Data

Transmembrane potentials were calculated using the Gromacs tool *gmx potential* for 100 slides along the normal path to the membrane. The reference was set to one edge of the pore axis, with the net charge assumed to be zero.

MDAnalysis 2.7,^69^ Matplotlip 3.8.4,^70^ Numpy 1.26.1,^71^ and Scipy 1.13 ^72^ were used for the analysis of ion position during permeation, 1D and 2D ion occupancy, and conductance. To detect ion permeation through the channel, periodic boundary effects were removed, and all protein structures were fitted to the first protein structure as a reference. On this trajectory, a virtual cylinder was defined, centered at the Cα atom of S102, with a radius of 2 nm and a height of 5 nm for each monomer. For tunnel calculations using Caver 3.0,^73^ the central gate residues (C1C2: S102, E129, N297; ChR2: S63, E90, N258; iChloC: S63, R90, and N258) were selected as the starting point, with a probe radius of 1.1 Å, a shell radius of 3 Å, a shell depth of 4 Å, and a tunnel-clustering threshold of 3.5 Å. The number of first hydration water molecules within a sphere of 3.5 Å centered on K^+^ or within a sphere of 3.2 Å centered on Na^+^ ^74^ were calculated using MDAnalysis 2.7.^69^ VMD 1.9.4 ^75^ was used for ion scatter plot. PyMOL 3.0.3 ^76^ was used for molecular visualization and movie generation.

## Supplementary Material





## Data Availability

The simulation run input files, comprising the starting configuration and all necessary parameters for performing the MD simulations are deposited in Zenodo under https://doi.org/10.5281/zenodo.15532847 .

## Notes

Single-cation conduction mechanism (MP4)
Additional details, including figures and tables, for the CompEL-MD system, water occupancy, ion conduction, pore opening, system stability, ion binding sites, cpH-MD, simulation systems, and their related references (PDF)

## References

[1] Nagel G., Ollig D., Fuhrmann M., Kateriya S., Mustl A. M., Bamberg E., Hegemann P. (2002). Channelrhodopsin-1: A light-gated
proton channel in green algae. Science.

[2] Zhang F., Vierock J., Yizhar O., Fenno L. E., Tsunoda S., Kianianmomeni A., Prigge M., Berndt A., Cushman J., Polle J. (2011). The Microbial Opsin
Family of Optogenetic Tools. Cell.

[3] Pimentel D., Donlea J. M., Albot C. B. T., Ong S. M. S., Hurston A. J. F. T., Miesenböck G. (2016). Operation
of a homeostatic sleep switch. Nature.

[4] Yang Y. Y., Wu M. Z., Vázquez-Guardado A., Wegener A. J., Grajales-Reyes J. G., Deng Y. J., Wang T. Y., Avila R., Moreno J. A., Minkowicz S. (2021). Wireless multilateral
devices for optogenetic studies of individual and social behaviors. Nat. Neurosci..

[5] Hsueh B., Chen R., Jo Y., Tang D., Raffiee M., Kim Y. S., Inoue M., Randles S., Ramakrishnan C., Patel S. (2023). Cardiogenic
control of affective behavioural state. Nature.

[6] Iyer S. M., Vesuna S., Ramakrishnan C., Huynh K., Young S., Berndt A., Lee S. Y., Gorini C. J., Deisseroth K., Delp S. L. (2016). Optogenetic and
chemogenetic strategies for sustained
inhibition of pain. Sci. Rep..

[7] Sahel J. A., Boulanger-Scemama E., Pagot C., Arleo A., Galluppi F., Martel J. N., Esposti S. D., Delaux A., Aubert J. B. D., de
Montleau C. (2021). Partial recovery of
visual function in a blind patient after optogenetic therapy. Nat. Med..

[8] Hernandez V. H., Gehrt A., Reuter K., Jing Z., Jeschke M., Schulz A. M., Hoch G., Bartels M., Vogt G., Garnham C. W. (2014). Optogenetic
stimulation of the auditory pathway. J. Clin.
Invest..

[9] Valverde S., Vandecasteele M., Piette C., Derousseaux W., Gangarossa G., Aristieta Arbelaiz A., Touboul J., Degos B., Venance L. (2020). Deep brain
stimulation-guided optogenetic rescue of
parkinsonian symptoms. Nat. Commun..

[10] Tønnesen J., Sorensen A. T., Deisseroth K., Lundberg C., Kokaia M. (2009). Optogenetic
control of epileptiform activity. Proc. Natl.
Acad. Sci. U.S.A..

[11] Nagel G., Szellas T., Huhn W., Kateriya S., Adeishvili N., Berthold P., Ollig D., Hegemann P., Bamberg E. (2003). Channelrhodopsin-2,
a directly light-gated cation-selective membrane channel. Proc. Natl. Acad. Sci. U.S.A..

[12] Klapoetke N. C., Murata Y., Kim S. S., Pulver S. R., Birdsey-Benson A., Cho Y. K., Morimoto T. K., Chuong A. S., Carpenter E. J., Tian Z. J. (2014). Independent
optical excitation of distinct neural populations. Nat. Methods.

[13] Govorunova E. G., Sineshchekov O. A., Li H., Janz R., Spudich J. L. (2013). Characterization
of a Highly Efficient Blue-shifted Channelrhodopsin from the Marine
Alga. J. Biol. Chem..

[14] Govorunova E. G., Gou Y. Y., Sineshchekov O. A., Li H., Lu X. Y., Wang Y. M., Brown L. S., St-Pierre F., Xue M. S., Spudich J. L. (2022). Kalium channelrhodopsins are natural
light-gated potassium channels that mediate optogenetic inhibition. Nat. Neurosci..

[15] Vierock J., Peter E., Grimm C., Rozenberg A., Chen I. W., Tillert L., Scalise A. G. C., Casini M., Augustin S., Tanese D. (2022). WiChR, a highly potassium-selective
channelrhodopsin for low-light one- and two-photon inhibition of excitable
cells. Sci. Adv..

[16] Govorunova E. G., Sineshchekov O. A., Janz R., Liu X. Q., Spudich J. L. (2015). Natural
light-gated anion channels: A family of microbial rhodopsins for advanced
optogenetics. Science.

[17] Berndt A., Lee S. Y., Ramakrishnan C., Deisseroth K. (2014). Structure-Guided
Transformation of Channelrhodopsin into a Light-Activated Chloride
Channel. Science.

[18] Wietek J., Beltramo R., Scanziani M., Hegemann P., Oertner T. G., Wiegert J. S. (2015). An improved chloride-conducting
channelrhodopsin for
light-induced inhibition of neuronal activity in vivo. Sci. Rep..

[19] Fernandez
Lahore R. G., Pampaloni N. P., Peter E., Heim M. M., Tillert L., Vierock J., Oppermann J., Walther J., Schmitz D., Owald D. (2022). Calcium-permeable
channelrhodopsins for the photocontrol of calcium signalling. Nat. Commun..

[20] Lórenz-Fonfría V. A., Resler T., Krause N., Nack M., Gossing M., von Mollard G. F., Bamann C., Bamberg E., Schlesinger R., Heberle J. (2013). Transient protonation changes in channelrhodopsin-2
and their relevance to channel gating. Proc.
Natl. Acad. Sci. U.S.A..

[21] Shibata K., Oda K., Nishizawa T., Hazama Y., Ono R., Takaramoto S., Bagherzadeh R., Yawo H., Nureki O., Inoue K., Akiyama H. (2023). Twisting and Protonation of Retinal Chromophore Regulate
Channel Gating of Channelrhodopsin C1C2. J.
Am. Chem. Soc..

[22] Kuhne J., Vierock J., Tennigkeit S. A., Dreier M. A., Wietek J., Petersen D., Gavriljuk K., El-Mashtoly S. F., Hegemann P., Gerwert K. (2019). Unifying photocycle
model for light
adaptation and temporal evolution of cation conductance in channelrhodopsin-2. Proc. Natl. Acad. Sci. U.S.A..

[23] Schneider F., Gradmann D., Hegemann P. (2013). Ion Selectivity
and Competition in
Channelrhodopsins. Biophys. J..

[24] Kato H. E., Zhang F., Yizhar O., Ramakrishnan C., Nishizawa T., Hirata K., Ito J., Aita Y., Tsukazaki T., Hayashi S. (2012). Crystal
structure of
the channelrhodopsin light-gated cation channel. Nature.

[25] Volkov O., Kovalev K., Polovinkin V., Borshchevskiy V., Bamann C., Astashkin R., Marin E., Popov A., Balandin T., Willbold D. (2017). Structural insights
into ion conduction by channelrhodopsin 2. Science.

[26] Kim Y. S., Kato H. E., Yamashita K., Ito S., Inoue K., Ramakrishnan C., Fenno L. E., Evans K. E., Paggi J. M., Dror R. O. (2018). Crystal structure of
the natural anion-conducting channelrhodopsin ACR1. Nature.

[27] Kishi K. E., Kim Y. S., Fukuda M., Inoue M., Kusakizako T., Wang P. Y., Ramakrishnan C., Byrne E. F. X., Thadhani E., Paggi J. M. (2022). Structural
basis for channel conduction in the pump-like channelrhodopsin ChRmine. Cell.

[28] Rozenberg A., Kaczmarczyk I., Matzov D., Vierock J., Nagata T., Sugiura M., Katayama K., Kawasaki Y., Konno M., Nagasaka Y. (2022). Rhodopsin-bestrophin
fusion proteins from unicellular
algae form gigantic pentameric ion channels. Nat. Struct. Mol. Biol..

[29] Tajima S., Kim Y. S., Fukuda M., Jo Y., Wang P. Y., Paggi J. M., Inoue M., Byrne E. F. X., Kishi K. E., Nakamura S. (2023). Structural basis for
ion selectivity in potassium- selective channelrhodopsins. Cell.

[30] Morizumi T., Kim K., Li H., Nag P., Dogon T., Sineshchekov O. A., Wang Y. M., Brown L. S., Hwang S., Sun H. (2025). Structural insights
into light-gating of potassium-selective channelrhodopsin. Nat. Commun..

[31] Oda K., Nomura T., Nakane T., Yamashita K., Inoue K., Ito S., Vierock J., Hirata K., Maturana A. D., Katayama K. (2021). Time-resolved serial
femtosecond crystallography reveals early structural changes in channelrhodopsin. eLife.

[32] Mulder M., Hwang S., Broser M., Brünle S., Skopintsev P., Schattenberg C., Schnick C., Hartmann S., Church J., Schapiro I. (2025). Structural Insights
Into the Opening Mechanism of C1C2 Channelrhodopsin. J. Am. Chem. Soc..

[33] Takemoto M., Kato H. E., Koyama M., Ito J., Kamiya M., Hayashi S., Maturana A. D., Deisseroth K., Ishitani R., Nureki O. (2015). Molecular Dynamics of Channelrhodopsin
at the Early Stages of Channel Opening. PLoS
One.

[34] Kaufmann J. C. D., Krause B. S., Adam S., Ritter E., Schapiro I., Hegemann P., Bartl F. J. (2020). Modulation
of Light Energy Transfer from Chromophore to Protein in the Channelrhodopsin
ReaChR. Biophys. J..

[35] Kuhne J., Eisenhauer K., Ritter E., Hegemann P., Gerwert K., Bartl F. (2015). Early Formation of the Ion-Conducting Pore in Channelrhodopsin-2. Angew. Chem., Int. Ed..

[36] Schneider F., Grimm C., Hegemann P. (2015). Biophysics
of Channelrhodopsin. Annu. Rev. Biophys..

[37] Ives C. M., Thomson N. J., Zachariae U. (2023). A cooperative
knock-on mechanism
underpins Ca2+-selective cation permeation in TRPV channels. J. Gen. Physiol..

[38] Saponaro A., Bauer D., Giese M. H., Swuec P., Porro A., Gasparri F., Sharifzadeh A. S., Chaves-Sanjuan A., Alberio L., Parisi G. (2021). Gating
movements and
ion permeation in HCN4 pacemaker channels. Mol.
Cell.

[39] Biedermann J., Braunbeck S., Plested A. J. R., Sun H. (2021). Nonselective cation
permeation in an AMPA-type glutamate receptor. Proc. Natl. Acad. Sci. U.S.A..

[40] Silapetere A., Hwang S., Hontani Y., Lahore R. F. G., Balke J., Escobar F. V., Tros M., Konold P. E., Matis R., Croce R. (2022). Author
Correction: QuasAr Odyssey: the origin of fluorescence
and its voltage sensitivity in microbial rhodopsins. Nat. Commun..

[41] Gaiko O., Dempski R. E. (2013). Transmembrane Domain Three Contributes to the Ion Conductance
Pathway of Channelrhodopsin-2. Biophys. J..

[42] Zhang A. H., Yu H., Liu C. H., Song C. (2020). The Ca permeation mechanism of the
ryanodine receptor revealed by a multi-site ion model. Nat. Commun..

[43] Schackert, F. K. ; Biedermann, J. ; Abdolvand, S. ; Minniberger, S. ; Song, C. ; Plested, A. J. R. ; Carloni, P. ; Sun, H. Mechanism of Calcium Permeation in a Glutamate Receptor Ion Channel. J. Chem. Inf. Model. 2023 63 1293 10.1021/acs.jcim.2c01494. 36758214 PMC9976283

[44] Feldbauer K., Zimmermann D., Pintschovius V., Spitz J., Bamann C., Bamberg E. (2009). Channelrhodopsin-2
is a leaky proton pump. Proc. Natl. Acad. Sci.
U.S.A..

[45] Aho, N. ; Buslaev, P. ; Jansen, A. ; Bauer, P. ; Groenhof, G. ; Hess, B. Scalable Constant pH Molecular Dynamics in GROMACS. J. Chem. Theory Comput. 2022 18 6148 10.1021/acs.jctc.2c00516. 36128977 PMC9558312

[46] Ritter E., Stehfest K., Berndt A., Hegemann P., Bartl F. J. (2008). Monitoring
Light-induced Structural Changes of Channelrhodopsin-2 by UV–visible
and Fourier Transform Infrared Spectroscopy. J. Biol. Chem..

[47] Kamiya M., Kato H. E., Ishitani R., Nureki O., Hayashi S. (2013). Structural and spectral characterizations
of C1C2 channelrhodopsin and its mutants by molecular simulations. Chem. Phys. Lett..

[48] a Yang, T. ; Zhang, W. ; Cheng, J. ; Nie, Y. ; Xin, Q. ; Yuan, S. ; Dou, Y. Formation Mechanism of Ion Channel in Channelrhodopsin-2: Molecular Dynamics Simulation and Steering Molecular Dynamics Simulations. Int. J. Mol. Sci. 2019, 20 (15 3780 10.3390/ijms20153780. 31382458 PMC6695816

[49] Prignano L. A., Stevens M. J., Vanegas J. M., Rempe S. B., Dempski R. E. (2024). Metadynamics
simulations reveal mechanisms of Na+ and Ca2+ transport in two open
states of the channelrhodopsin chimera, C1C2. PLoS One.

[50] Köpfer D. A., Song C., Gruene T., Sheldrick G. M., Zachariae U., de Groot B. L. (2014). Ion permeation in
K channels occurs by direct Coulomb knock-on. Science.

[51] Oh S., Marinelli F., Zhou W. C., Lee J., Choi H. J., Kim M., Faraldo-Gomez J. D., Hite R. K. (2022). Differential ion
dehydration energetics explains selectivity in the non-canonical lysosomal
K channel TMEM175. eLife.

[52] Zheng W., Shen C., Wang L. F., Rawson S., Xie W. J., Nist-Lund C., Wu J., Shen Z. F., Xia S. Y., Holt J. R. (2022). pH
regulates potassium conductance and drives a constitutive
proton current in human TMEM175. Sci. Adv..

[53] Kopec W., Köpfer D. A., Vickery O. N., Bondarenko A. S., Jansen T. L. C., de Groot B. L., Zachariae U. (2018). Direct knock-on
of desolvated ions governs strict ion selectivity in K channels. Nat. Chem..

[54] Liu H., Biedermann J., Sun H. (2024). Atomistic Mechanism of Cation Permeation
and Pore Voltage Sensitivity in Cyclic Nucleotide-Gated CNGA1 Ion
Channel. bioRxiv.

[55] Shi C. W., He Y., Hendriks K., de Groot B. L., Cai X. Y., Tian C. L., Lange A., Sun H. (2018). A single NaK channel conformation
is not enough for non-selective ion conduction. Nat. Commun..

[56] Gradmann D., Berndt A., Schneider F., Hegemann P. (2011). Rectification of the
Channelrhodopsin Early Conductance. Biophys.
J..

[57] Abraham M. J., Murtola T., Schulz R., Páll S., Smith J. C., Hess B., Lindahl E. (2015). GROMACS: High performance
molecular simulations through multi-level parallelism from laptops
to supercomputers. SoftwareX.

[58] Huang J., Rauscher S., Nawrocki G., Ran T., Feig M., de Groot B. L., Grubmüller H., MacKerell A. D. (2017). CHARMM36m:
an improved force field for folded and intrinsically disordered proteins. Nat. Methods.

[59] Tajkhorshid E., Paizs B., Suhai S. (1997). Conformational
effects on the proton affinity of the Schiff base in bacteriorhodopsin:
A density functional study. J. Phys. Chem. B.

[60] Jo S., Kim T., Iyer V. G., Im W. (2008). CHARMM-GUI: a web-based graphical
user interface for CHARMM. J. Comput. Chem..

[61] Sali A., Blundell T. L. (1993). Comparative Protein
Modelling by Satisfaction of Spatial
Restraints. J. Mol. Biol..

[62] Jorgensen W. L., Chandrasekhar J., Madura J. D., Impey R. W., Klein M. L. (1983). Comparison
of Simple Potential Functions for Simulating Liquid Water. J. Chem. Phys..

[63] Bussi G., Donadio D., Parrinello M. (2007). Canonical
sampling through velocity
rescaling. J. Chem. Phys..

[64] Berendsen H. J. C., Postma J. P. M., Vangunsteren W. F., Dinola A., Haak J. R. (1984). Molecular-Dynamics
with Coupling to an External Bath. J. Chem.
Phys..

[65] Parrinello M., Rahman A. (1981). Polymorphic Transitions in Single-Crystals - a New
Molecular-Dynamics Method. J. Appl. Phys..

[66] Kutzner C., Grubmüller H., de Groot B. L., Zachariae U. (2011). Computational
Electrophysiology: The Molecular Dynamics of Ion Channel Permeation
and Selectivity in Atomistic Detail. Biophys.
J..

[67] Darden T., York D., Pedersen L. (1993). Particle Mesh Ewald
- an N.Log­(N)
Method for Ewald Sums in Large Systems. J. Chem.
Phys..

[68] Hess B., Bekker H., Berendsen H. J. C., Fraaije J. G. E. M. (1997). LINCS: A linear
constraint solver for molecular simulations. J. Comput. Chem..

[69] Michaud-Agrawal N., Denning E. J., Woolf T. B., Beckstein O. (2011). MDAnalysis:
A Toolkit for the Analysis of Molecular Dynamics Simulations. J. Comput. Chem..

[70] Hunter J. D. (2007). Matplotlib:
A 2D graphics environment. Comput. Sci. Eng..

[71] Harris C. R., Millman K. J., van der Walt S. J., Gommers R., Virtanen P., Cournapeau D., Wieser E., Taylor J., Berg S., Smith N. J. (2020). Array programming with NumPy. Nature.

[72] Virtanen P., Gommers R., Oliphant T. E., Haberland M., Reddy T., Cournapeau D., Burovski E., Peterson P., Weckesser W., Bright J. (2020). SciPy 1.0: fundamental
algorithms for scientific computing in Python. Nat. Methods.

[73] Chovancova E., Pavelka A., Benes P., Strnad O., Brezovsky J., Kozlikova B., Gora A., Sustr V., Klvana M., Medek P. (2012). CAVER 3.0: A Tool for
the Analysis of Transport Pathways
in Dynamic Protein Structures. PLoS Comput.
Biol..

[74] Rowley C. N., Roux B. (2012). The Solvation Structure of Na^+^ and K^+^ in Liquid
Water Determined from High Level *ab Initio* Molecular
Dynamics Simulations. J. Chem. Theory Comput..

[75] Humphrey W., Dalke A., Schulten K. (1996). VMD: Visual
molecular dynamics. J. Mol. Graphics.

[76] Schrodinger, L. L. C. The PyMOL Molecular Graphics System, Version 1.8, 2015.

