# Supplementary material for: Pore-Opening and Ion-Conduction Mechanism in Channelrhodopsins C1C2, ChR2, and iChloC by Computational Electrophysiology and Constant-pH Simulations
Source: J Chem Inf Model. 2025 May 29;65(11):5649–61. doi: 10.1021/acs.jcim.5c00356 (PMC12152938; doi:10.1021/acs.jcim.5c00356)
Supplement: Supplementary file 2 [file ci5c00356_si_002.pdf]

Supporting Information for

Pore-Opening and Ion-Conduction Mechanism in Channelrhodopsins C1C2, ChR2 and iChloC by Computational Electrophysiology and Constant-pH Simulations

Songhwan Hwang[1,2], Tillmann Utesch[1], Caspar Schattenberg[1], Johannes Vierock[3], Han Sun[1,4,]*

[1]Research Unit of Structural Chemistry & Computational Biophysics, Leibniz-Forschungsinstitut für Molekulare Pharmakologie, Berlin 13125, Germany.

[2]Institute of Biology, Department of Experimental Biophysics, Humboldt-Universität zu Berlin, Berlin 10115, Germany.

[3]Neuroscience Research Centre, Charité Berlin, Berlin, Germany.

[4]Institute of Chemistry, Technische Universität Berlin, Berlin 10623, Germany.

*Corresponding author: hsun@fmp-berlin.de

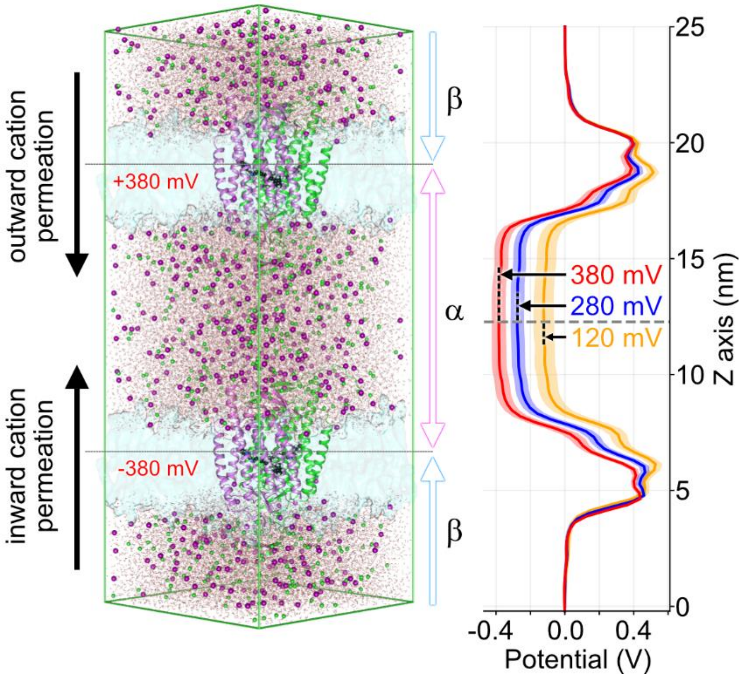

Fig. S1. Computational electrophysiology (CompEL)-MD simulations of C1C2. (Left) CompEL-MD simulation setup of C1C2, where the protein structure is depicted as a cartoon model, with the protonated retinylidene Schiff base represented as black spheres. The lipid bilayer is illustrated as a cyan surface, while water molecules are shown as red and white stick models. K^+ and Cl^- ions are shown as purple and green spheres, respectively. (Right) Membrane potentials of C1C2 with fixed charge imbalances of 2*e*, 4*e*, and 6*e* (corresponding to about 120 mV, 280 mV, and 380 mV) between the α and β compartments.

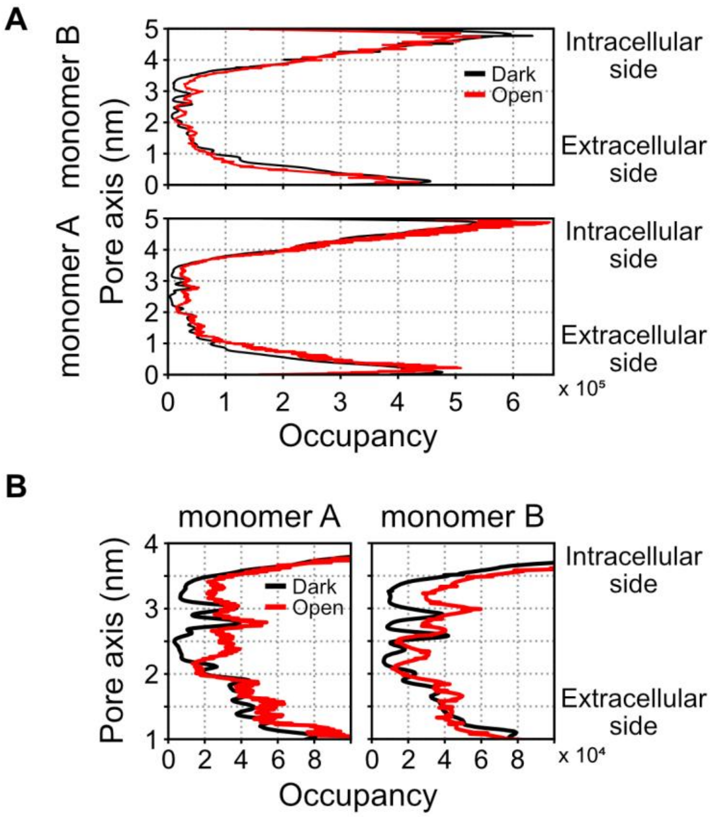

Fig. S2. Water occupancy in the pore of the dark and open states of C1C2. (A) Cumulative water occupancy along the pore in the dark (black) and open (red) states of C1C2. A cylinder with a 2-nm radius, centered at the Cα position of the central gate residue S102, was defined for this analysis. (B) Zoom-in view of the pore region shown in panel A.

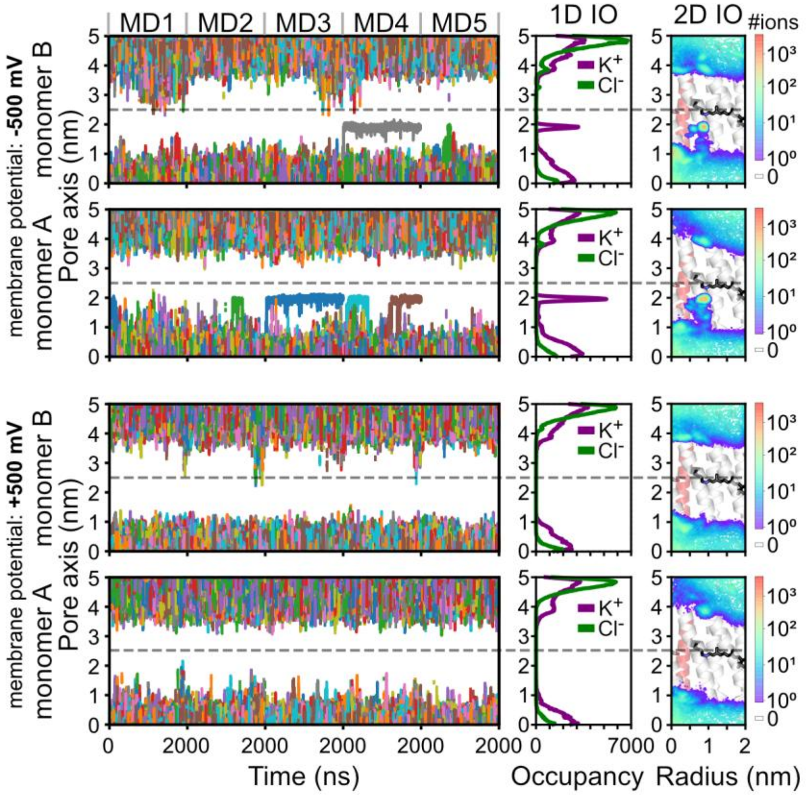

Fig. S3. Absence of cation permeation in dark-state C1C2 at -500 and +500 mV. (Left) Traces of K^+ ions within the pore of C1C2, with the central gate indicated as a dashed line. (Middle) Cumulative one-dimensional ion occupancy along the pore-axis. (Right) 2D K^+ density within the pore region mapped onto the dark-state structure of C1C2. The protonated retinylidene Schiff base shown in black, and TM2 colored in red.

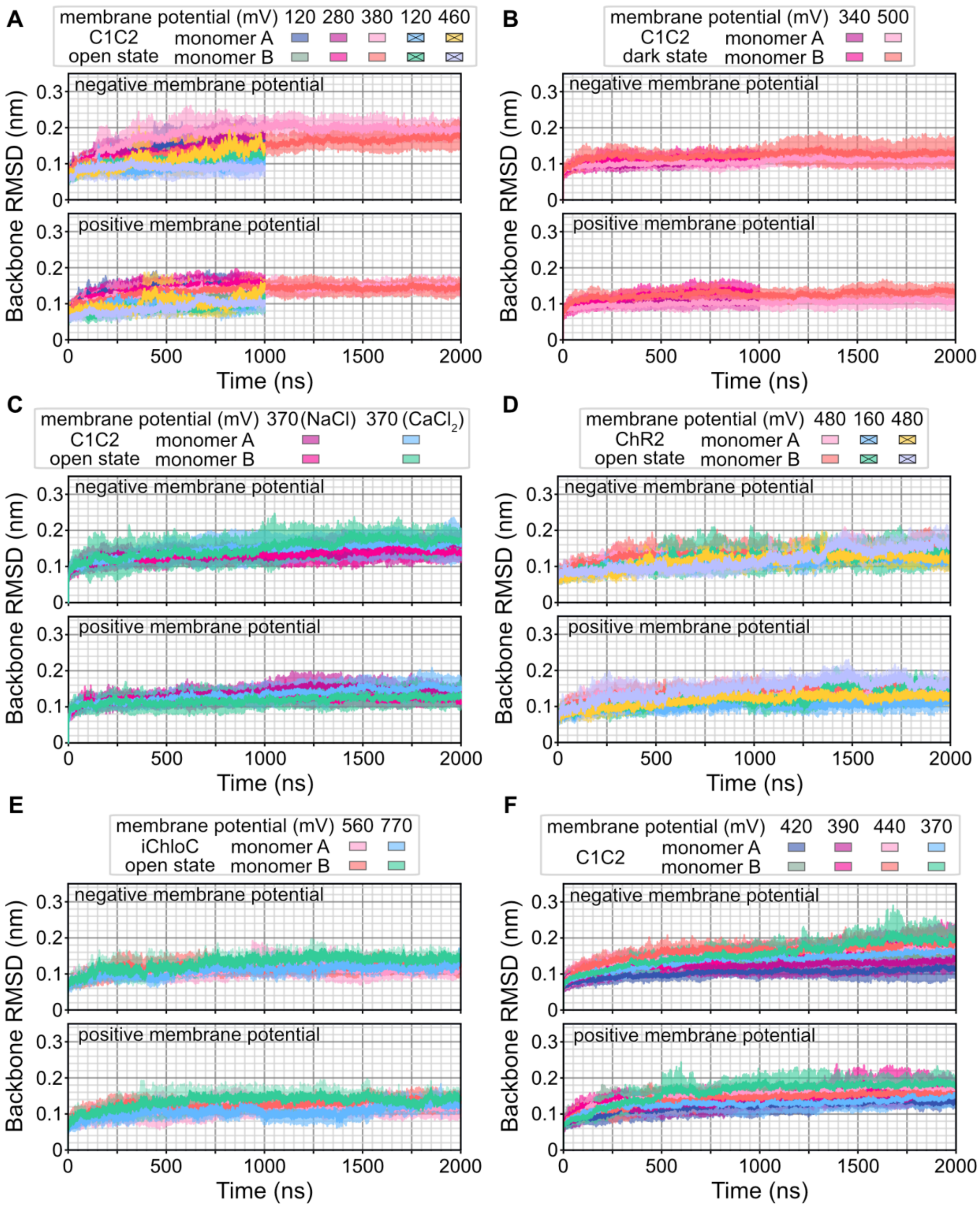

Fig. S4. Backbone root mean square deviations (RMSDs) of C1C2, ChR2, and iChloC. (A,B) Backbone RMSDs of monomer A and B for the open and dark states of C1C2 under negative and positive membrane potentials. (B) Prolonged CompEL-MD simulations starting from the inward-

permeating open-state C1C2 structure (obtained from the final snapshot of a 2-μs CompEL-MD simulation) are highlighted by a box with a cross. (C) CompEL-MD simulations initiated from the inward-permeating, open-state C1C2 structure obtained from the final snapshot of a previous study[1], with solvation in either NaCl or $CaCl_2$ replacing KCl. (D,E) Backbone RMSDs of monomers A and B for the open-states of (D) ChR2 and (E) iChloC under negative and positive membrane potentials. Prolonged CompEL-MD simulations starting from the inward-permeating open-state ChR2 structure (obtained from the final snapshot of a 2-μs CompEL-MD simulation) at -480 mV are highlighted by diagonal lines. (F) Backbone RMSDs of C1C2 with different protonation states: protonated D195 and D292 (420 mV); protonated E162 (390 mV); protonated E162 and D292 (440 mV); all deprotonated (370 mV). The slight differences in membrane voltages between the models result from the change in electrostatics in the proteins induced by protonation changes. The average RMSDs are represented as solid lines, while standard deviations are shown as shaded regions.

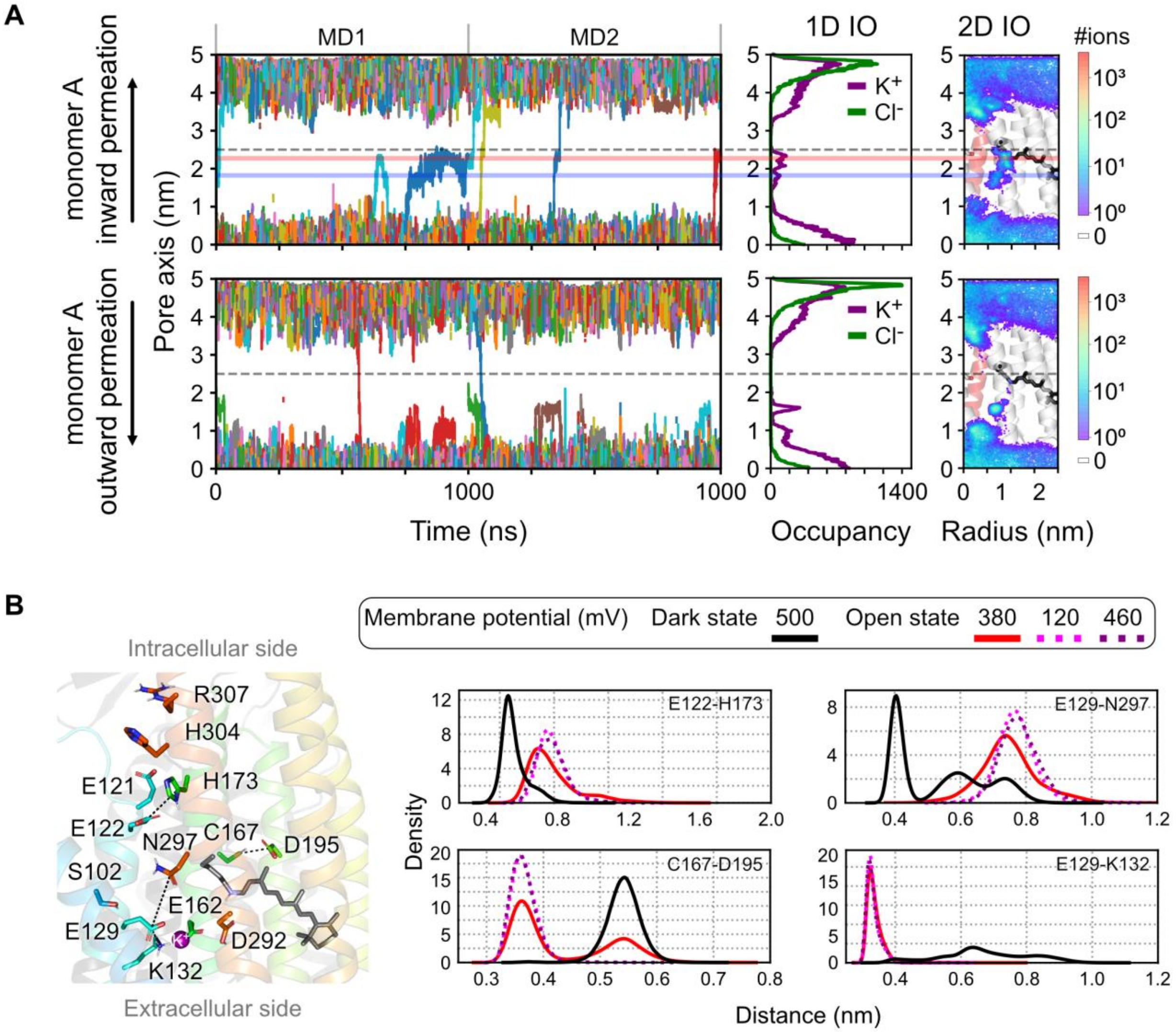

Fig. S5. Cation permeation in prolonged CompEL-MD simulations of the open-state C1C2. (A) Inward and outward K^+ permeation in C1C2. (Left) Traces of K^+ passing through the pore of C1C2. (Middle) Cumulative one-dimensional ion occupancy along the pore-axis. (Right) 2D K^+ density within the pore mapped onto the open-state C1C2 structure with the protonated retinylidene Schiff base shown in black and TM2 in red. Two major K^+ binding sites, site 1 and site 2, are indicated by semi-transparent lines in blue and red, respectively. (B) Comparison of distance distributions between residue pairs in the dark state at 500 mV (black), the open state at

380 mV (red), and the prolonged open state at 120 mV (magenta) and 460 mV (purple). All distance distributions were calculated for monomer A. For distance calculation, the following atoms were considered: Sγ of Cys, Cγ of Asp, Cδ of Glu, Cγ of His, Nζ of Lys, and Cγ of Asn.

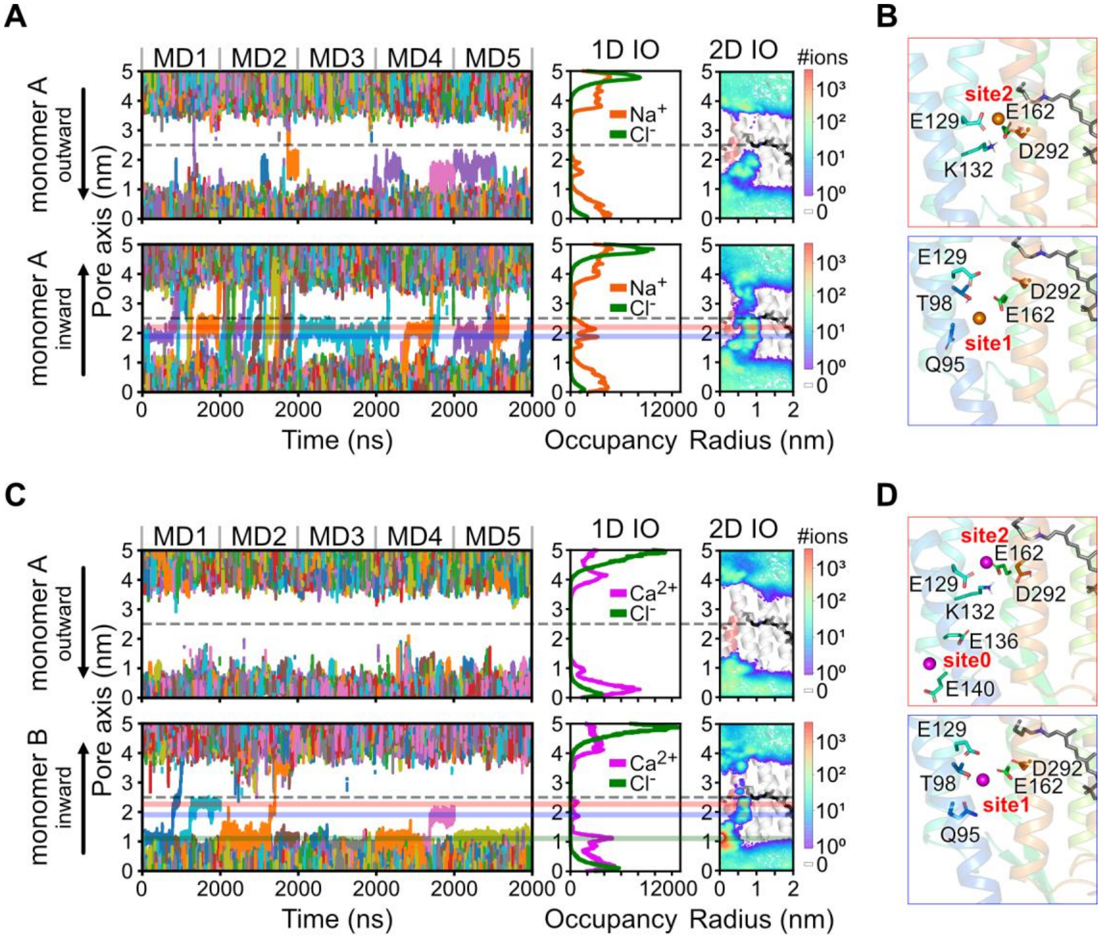

Fig. S6. Na^{+}/Ca^{2+} permeation of the CompEL-MD simulations of open-state C1C2. Inward and outward (A) Na^{+} and (C) Ca^{2+} permeations in C1C2. (Left) Traces of Na^{+}/Ca^{2+} passing through the pore of C1C2. (Middle) Cumulative one-dimensional ion occupancy along the pore-axis. (Right) 2D Na^{+}/Ca^{2+} density within the pore region, mapped onto the open-state structure of C1C2. (A, C) Two major K^{+}/Ca^{2+} binding sites, site 1 and site 2, are indicated by semi-transparent lines in blue and red, respectively. An additional Ca^{2+} binding site, site 0, is indicated by a semi-transparent green line. Major binding sites of (B) Na^{+} and (D) Ca^{2+} in the pore of C1C2. Ion binding residues are shown as sticks, and Na^{+}/Ca^{2+} ions are presented as spheres. All CompEL-MD simulations were initiated from the inward-permeating, open-state C1C2 structure obtained from the final snapshot of a previous study[1], with solvation in either NaCl or $CaCl_2$.

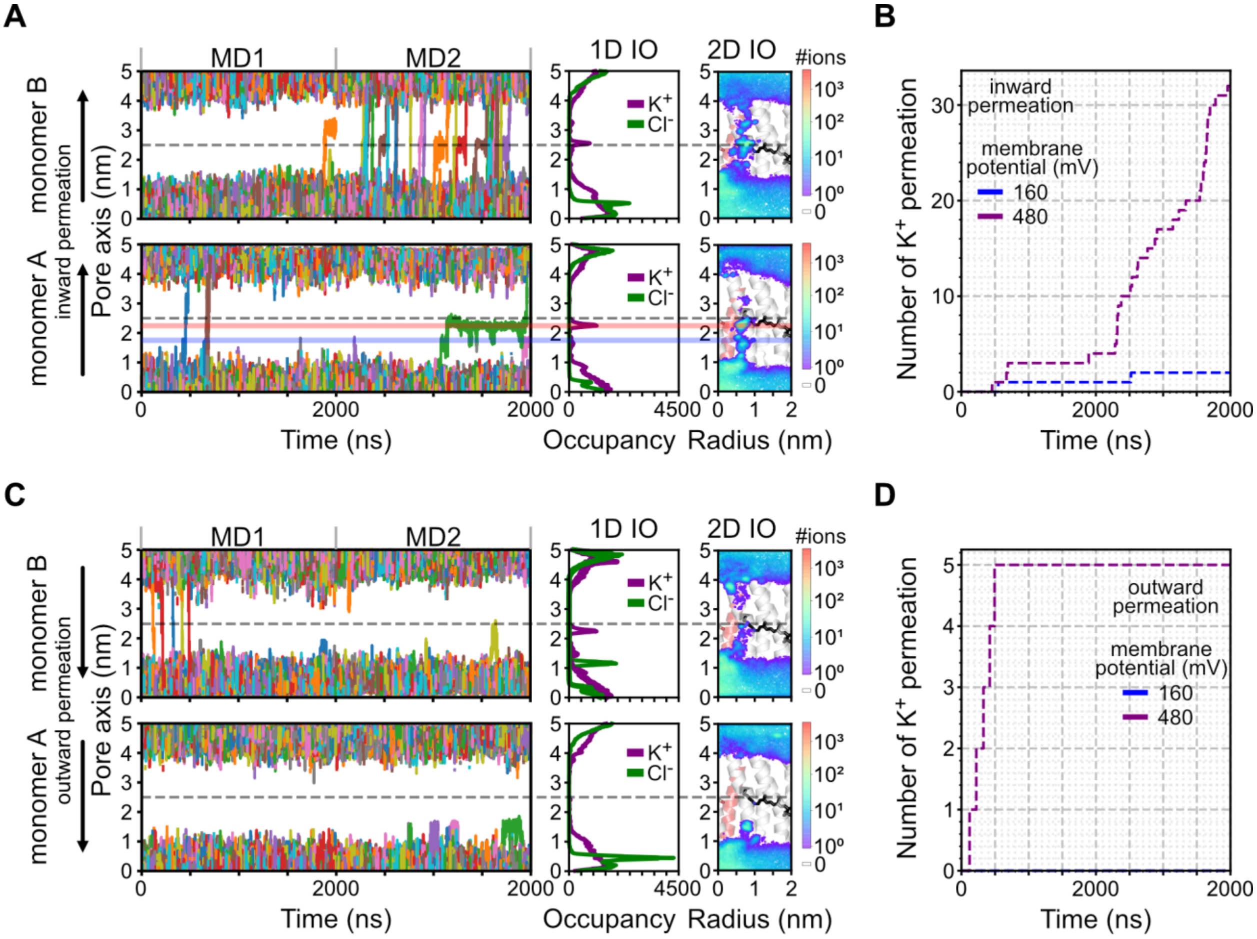

Fig. S7. Cation permeation in prolonged CompEL-MD simulations of open-state ChR2 at ±480 mV. (A) Inward and (C) outward K^+ permeations in ChR2. (Left) Traces of K^+ passing through the pore in ChR2. (Middle) Cumulative one-dimensional ion occupancy along the pore-axis. (Right) 2D K^+ density within the pore region, mapped onto the open-state ChR2 structure. Two major K^+ binding sites, site 1 and site 2, are indicated by semi-transparent lines in blue and red, respectively. Cumulative number of (B) inward and (D) outward K^+ permeation events.

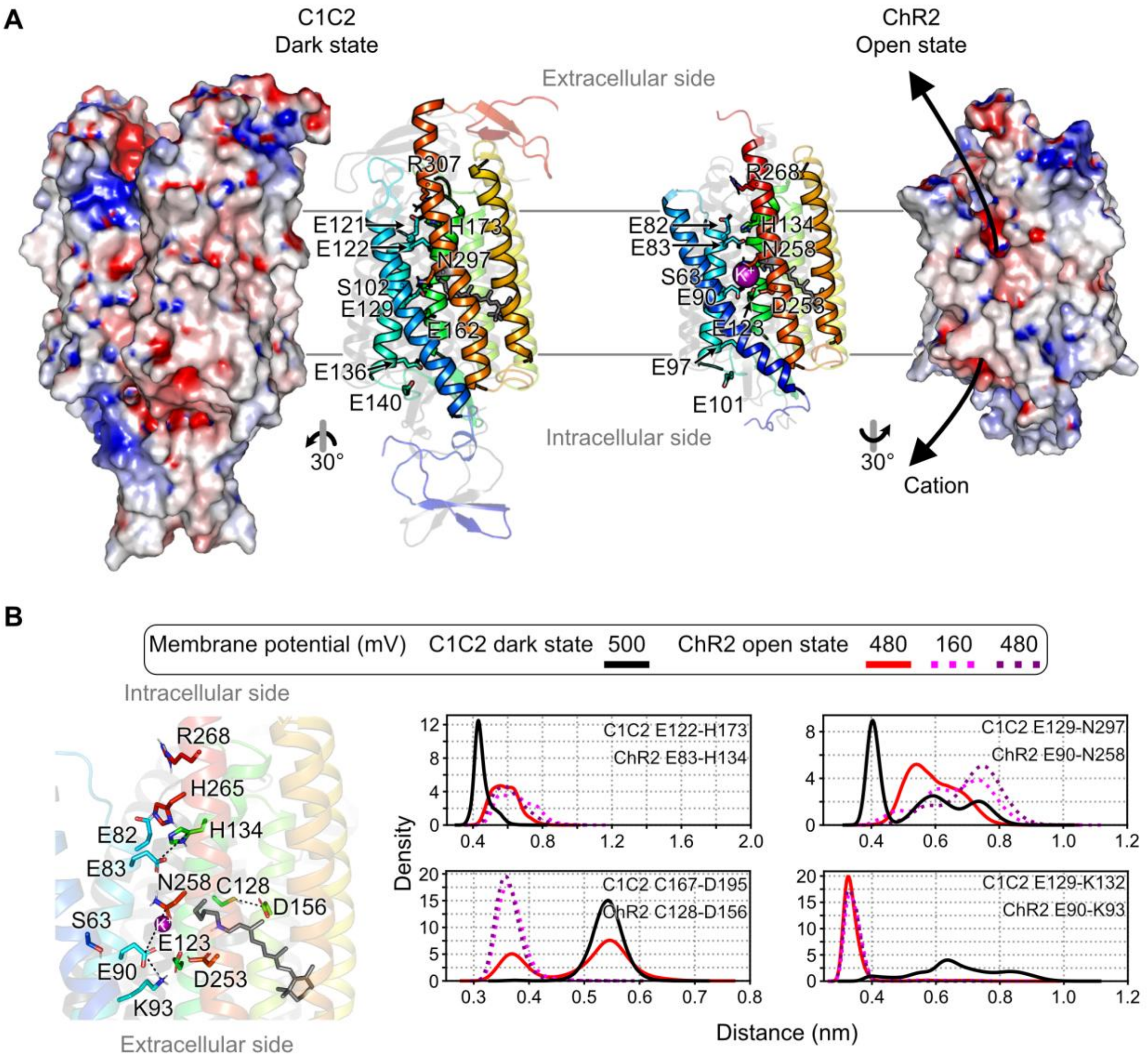

Fig. S8. Dark state of C1C2 and open states of ChR2. (A) Visualization of the dark state of C1C2 and open states of ChR2 using electrostatic potential maps and cartoon representations. Key residues critical for cation conduction (E83, E90, E97, E101, and E123 in ChR2) are depicted as sticks. The illustrated structures represent the final snapshots from one of five replicas. (B) Comparative analysis of distance distributions between residue pairs in dark-state C1C2 at -500 mV (black) and open-state ChR2 at -480 (solid red line), -160 mV (dotted magenta line), and prolonged open-state simulations at -480 mV (dotted purple line). All distance distributions were

calculated for monomer A in C1C2 and for monomer B in ChR2. All distance distributions were derived from inward-permeating channels across five 2-μs simulation replicas, with prolonged simulations replicated twice, each consisting of 2-μs funs. For distance calculation, the following atoms were considered: Sγ of Cys, Cγ of Asp, Cδ of Glu, Cγ of His, Nζ of Lys, and Cγ of Asn.

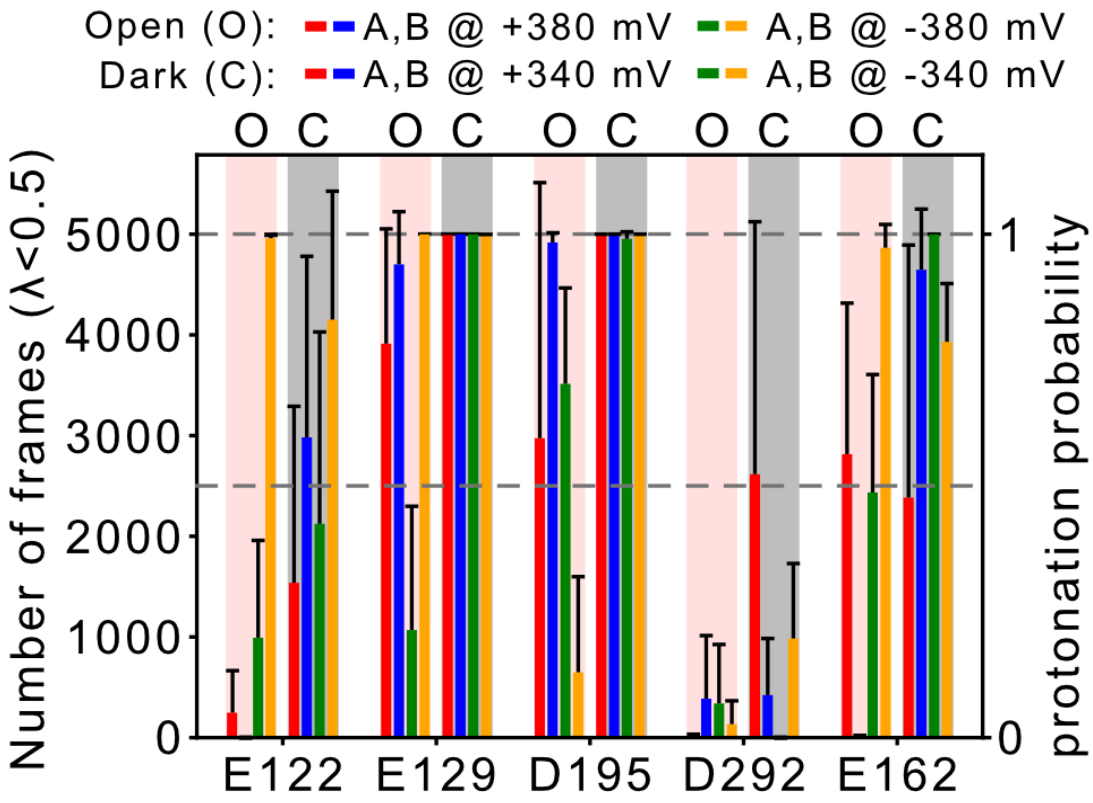

Fig. S9. Probing protonation states of key titratable residues. Titratable residues in the inner gate (E122), central gate (E129), DC pair (D195), and counterions (D292 and E162) showed variations in constant pH (cpH) - MD simulations. Starting structures were selected from the final snapshots of CompEL-MD simulations of the dark and open states of C1C2. A titratable residue in a snapshot taken every 100 ps of the cpH-MD simulations was considered protonated if $\lambda < 0.5$. The total occurrences of protonated residues across all 5000 frames were summed for the open (O or red shaded) and dark (closed, C or grey shaded) states and, for each subunit under positive (A: red; B: blue) and negative (A: green; B: yellow) voltages. Black bars represent the errors of the averages derived from three independent 500-ns cpH-MD simulations. The continuous reaction coordinate λ indicates the protonation state of titaratable residues. While λ=0 indicates the protonated, λ=1 corresponds to the deprotonated state for the shown acidic residues. This underlying energy function defines the behavior of intermediate states in this continuous model.

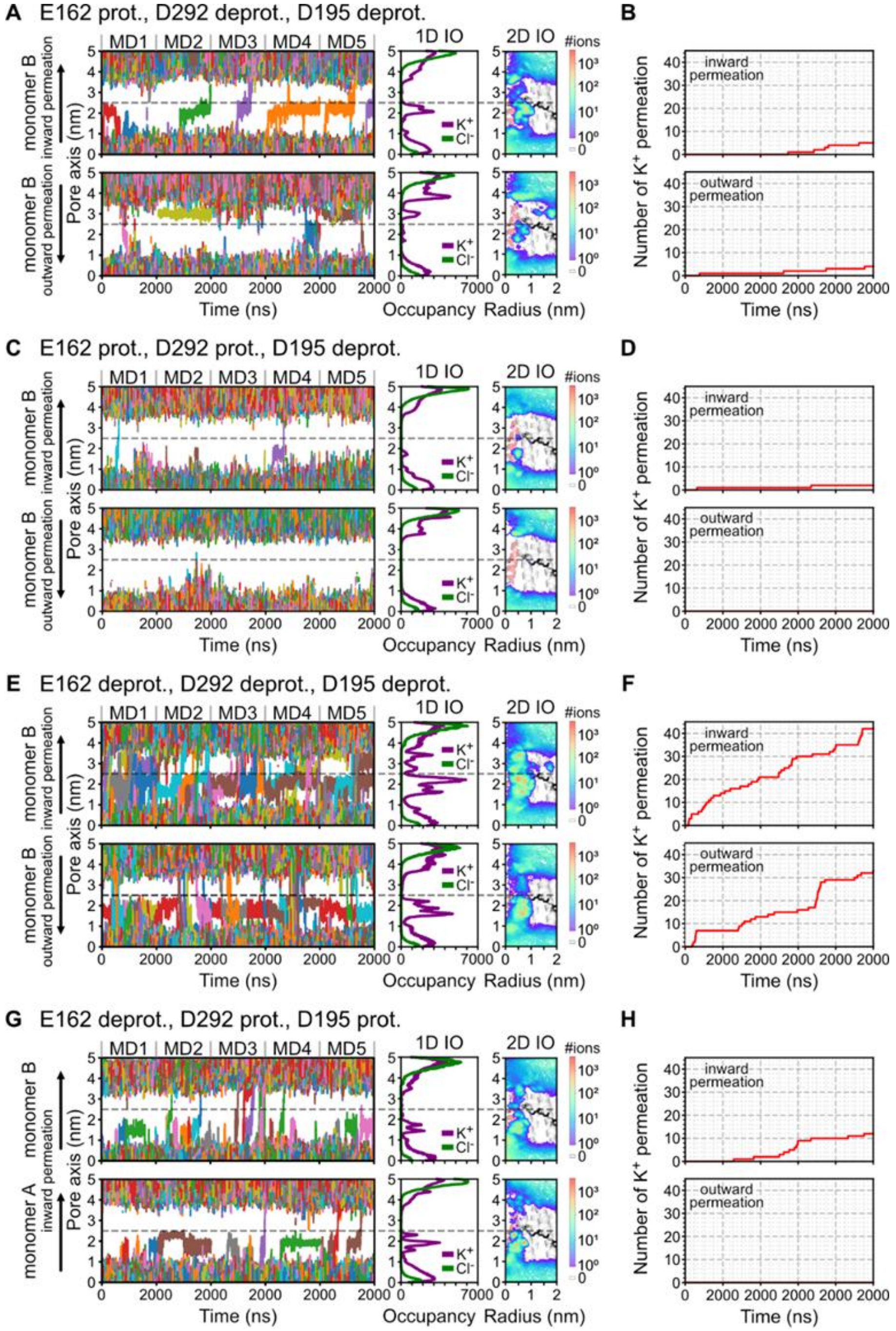

Fig. S10. Cation conduction in C1C2 with alternative protonation states. (A,C,E,G) (Left) Traces of K^+ ions within the C1C2 pore, confined to a 2-nm radius cylinder centered at the Cα position of the central gate residue S102 (grey dotted lines). (Middle) Cumulative one-dimensional ion occupancy along the pore axis. (Right) 2D K^+ density within the pore region, mapped onto the

open-state structure of C1C2. The protonated retinylidene Schiff base is shown as a black stick model, with TM2 colored in red. (B,D,F,H) Cumulative counts of inward and outward K^+ permeation events in C1C2.

Table S1. Assigned protonation states of residues in dark and open states for CompEL-MD simulations. Protonations were defined based on prior spectroscopic and electrophysiology experiments[2].

	Inner gate	Central gate	DC pair	Counterions	
C1C2	E122	E129	D195	E162	D292
Dark state	prot.	prot.	prot.	deprot.	deprot.
Open state	deprot.	deprot.	deprot.	deprot.	prot.
ChR2	E83	E90	D156	E123	D253
Open state	deprot.	deprot.	deprot.	deprot.	prot.
iChloC	E83	R90	N156	E123	D253
Open state	deprot.	-	-	deprot.	prot.

Table S2. Summary of CompEL-MD simulations for dark and open states of C1C2, open states of ChR2 and iChloC. *V*: membrane potential (mV), *Δq*: charge imbalance (*e*), *t*: individual simulation time (μs), *n*: the number of replicas, N_I: total inward permeations, N_O: total outward permeations, G_I: inward conductivity (pS) , G_O: outward conductivity (pS) , *Dim*: box size (X/Y/Z nm), $N_{cation/anion}$: the number of cation/anion, N_{water}: the number of water molecules, N_{POPC}: the number of POPC. All simulations were conducted at a KCl/NaCl/$CaCl_2$ concentration of 600 mM and at a temperature of 303 K. The prolonged CompEL-MD simulations were started from the inward permeating open-state structures of C1C2 and ChR2, derived from the end snapshots of the CompEL-MD simulations at -380 mV and -480 mV, respectively.

	V	*Δq*	*t*	*n*	N_I	N_O	G_I	G_O	*Dim*	$N_{cation/anion}$	N_{water}	N_{POPC}
C1C2 dark	343±36	4	1	5	0	0	0	0	9.9/9.9/25.9	626/614	56824	528
	503±30	6	2	5	0	0	0	0				
C1C2 open	122±55	2	1	5	0	0	0	0	9.9/9.9/25.3	618/598	55752	530
	277±36	4	1	5	8	1	0.9	0.1				
	384±37	6	2	5	24	1	1.0	0.0				
C1C2 open (prolonged)	116±9	2	1	2	1	1	0.7	0.7	9.9/9.9/26.5	654/634	58896	530
	462±8	6	1	2	4	2	0.7	0.4				
C1C2 open (NaCl)	365±18	6	2	5	45	5	1.9	0.2	9.9/9.9/26.2	650/626	58400	534
C1C2 open ($CaCl_2$)	370±22	6	2	5	2	0	0.1	0	9.9/9.9/26.1	440/856	58324	534
ChR2	477±26	6	2	5	18	0	0.6	0	8.9/8.9/21.2	368/380	34660	416
ChR2 (prolonged)	158±9	2	2	2	2	0	0.5	0	8.9/8.9/22.1	380/392	37374	416
	484±23	6	2	2	32	5	2.7	0.4				
iChloC	556±17	6	2	5	0	0	0	0	8.9/8.9/21.2	358/390	34060	418
	772±50	8	4	2	3	3	0.1	0.1				

Table S3. Summary of CompEL-MD simulations of C1C2 with varied protonation states of E162, D292, and D195. V: membrane potential (mV), N_I: total inward permeations, N_O: total outward permeations, G_I: inward conductivity (pS), G_O: outward conductivity (pS), *Dim*: box size (X/Y/Z nm), $N_{K/Cl}$: the number of K^+/Cl^-, N_{water}: the number of water molecules, N_{POPC}: the number of POPC. For each simulation setup of varied protonation states, 2 μs simulations were replicated five times at a KCl concentration of 600 mM and at a temperature of 303 K. Protonation (p) indicates a neutral state, while deprotonation (d) indicates a negatively charged state for the acidic residues. The slight differences in membrane voltages between the models result from the change in electrostatics in the proteins induced by protonation changes.

C1C2 protonation			V	N_I	N_O	G_I	G_O	*Dim*	$N_{K/Cl}$	N_{water}	N_{POPC}
E162	D292	D195									
d	p	d	384±37	24	1	1.01	0.04	9.9/9.9/25.3	618/598	55752	530
p	d	d	393±26	5	4	0.21	0.16	9.9/9.9/25.3	618/598	55786	530
p	p	d	444±27	2	0	0.07	0	9.9/9.9/25.3	614/598	55732	530
d	d	d	373±25	42	33	1.82	1.43	9.9/9.9/25.3	624/600	55794	530
d	p	p	420±36	12	0	0.46	0	9.9/9.9/25.3	614/598	55800	530

Table S4. Applied positional/dihedral angular restraints during energy minimization, equilibration simulations. Time step: Δt (fs), individual simulation time: t (ns), force constant (kJ/mol/nm) for position/angle harmornic restraints of backbone heavy atoms (k_{bb}), side-chain heavy atoms (k_{sc}), lipid head group (k_{head}), and lipid chirality and cis double bond ($k_{torsion}$).

	Δt	t	k_{bb}	k_{sc}	k_{head}	$k_{torsion}$
Energy minimization	-	-	4000	2000	1000	1000
Equilibration 1	1	0.25	4000	2000	1000	1000
Equilibration 2	1	0.25	2000	1000	400	400
Equilibration 3	1	0.25	1000	500	400	200
Equilibration 4	2	1	500	200	200	200
Equilibration 5	2	1	200	50	40	100
Equilibration 6	2	1	50	0	0	0
Equilibration 7	2	100	0	0	0	0

Movie S1 (separate file). K^+ translocation via the open-state C1C2. The MD trajectory from 1600 ns to 1640 ns of monomer A (green) and monomer B (grey) of the open state of C1C2 is shown. K^+, Cl^-, and water molecules are represented by purple, green, and cyan spheres, respectively. The key residues along the K^+ translocation pathway - (from the bottom to the top) E140, E136, K132, E162, E129, D292, S102, N297, E122, E121, H173, and R307 - are depicted as stick models. For clarity, POPC lipids are not shown.

References

(1) Mulder, M.; Hwang, S.; Broser, M.; Brünle, S.; Skopintsev, P.; Schattenberg, C.; Schnick, C.; Hartmann, S.; Church, J.; Schapiro, I.; et al. Structural Insights Into the Opening Mechanism of C1C2 Channelrhodopsin. *Journal of the American Chemical Society* **2024**, *147* (1), 1282-1290.
(2) (a) Lórenz-Fonfría, V. A.; Resler, T.; Krause, N.; Nack, M.; Gossing, M.; von Mollard, G. F.; Bamann, C.; Bamberg, E.; Schlesinger, R.; Heberle, J. Transient protonation changes in channelrhodopsin-2 and their relevance to channel gating. *P Natl Acad Sci USA* **2013**, *110* (14), E1273-E1281. (b) Kuhne, J.; Vierock, J.; Tennigkeit, S. A.; Dreier, M. A.; Wietek, J.; Petersen, D.; Gavriljuk, K.; El-Mashtoly, S. F.; Hegemann, P.; Gerwert, K. Unifying photocycle model for light adaptation and temporal evolution of cation conductance in channelrhodopsin-2. *Proc Natl Acad Sci U S A* **2019**, *116* (19), 9380-9389. (c) Hontani, Y.; Marazzi, M.; Stehfest, K.; Mathes, T.; van Stokkum, I. H. M.; Elstner, M.; Hegemann, P.; Kennis, J. T. M. Reaction dynamics of the chimeric channelrhodopsin C1C2. *Sci Rep-Uk* **2017**, *7*. (d) Takemoto, M.; Kato, H. E.; Koyama, M.; Ito, J.; Kamiya, M.; Hayashi, S.; Maturana, A. D.; Deisseroth, K.; Ishitani, R.; Nureki, O. Molecular Dynamics of Channelrhodopsin at the Early Stages of Channel Opening. *Plos One* **2015**, *10* (6). (e) Oda, K.; Nomura, T.; Nakane, T.; Yamashita, K.; Inoue, K.; Ito, S.; Vierock, J.; Hirata, K.; Maturana, A. D.; Katayama, K.; et al. Time-resolved serial femtosecond crystallography reveals early structural changes in channelrhodopsin. *Elife* **2021**, *10*.
